# Supplementary material for: SEPTIN2 suppresses an IFN-γ-independent, proinflammatory macrophage activation pathway
Source: Nat Commun. 2023 Nov 17;14:7441. doi: 10.1038/s41467-023-43283-2 (PMC10656488; doi:10.1038/s41467-023-43283-2)
Supplement: Supplementary file 1 — Supplementary Information [file 41467_2023_43283_MOESM1_ESM.pdf]

Supplementary Information

Title: SEPTIN2 suppresses IFN-γ-independent macrophage proinflammatory activation

Authors: Beibei Fu[#], Yan Xiong [#], Zhou Sha, Weiwei Xue, Binbin Xu, Shun Tan, Dong Guo, Feng Lin, Lulu Wang, Jianjian Ji, Yang Luo[*], Xiaoyuan Lin[*], and Haibo Wu[*]

[#] Equally Contributing Authors

[*]**Corresponding authors**: Haibo Wu, E-mail: hbwu023@cqu.edu.cn

Xiaoyuan Lin, E-mail: linxiaoyuan@zedat.fu-berlin.de

Yang Luo, E-mail: luoy@cqu.edu.cn

23 **Supplementary Figures**

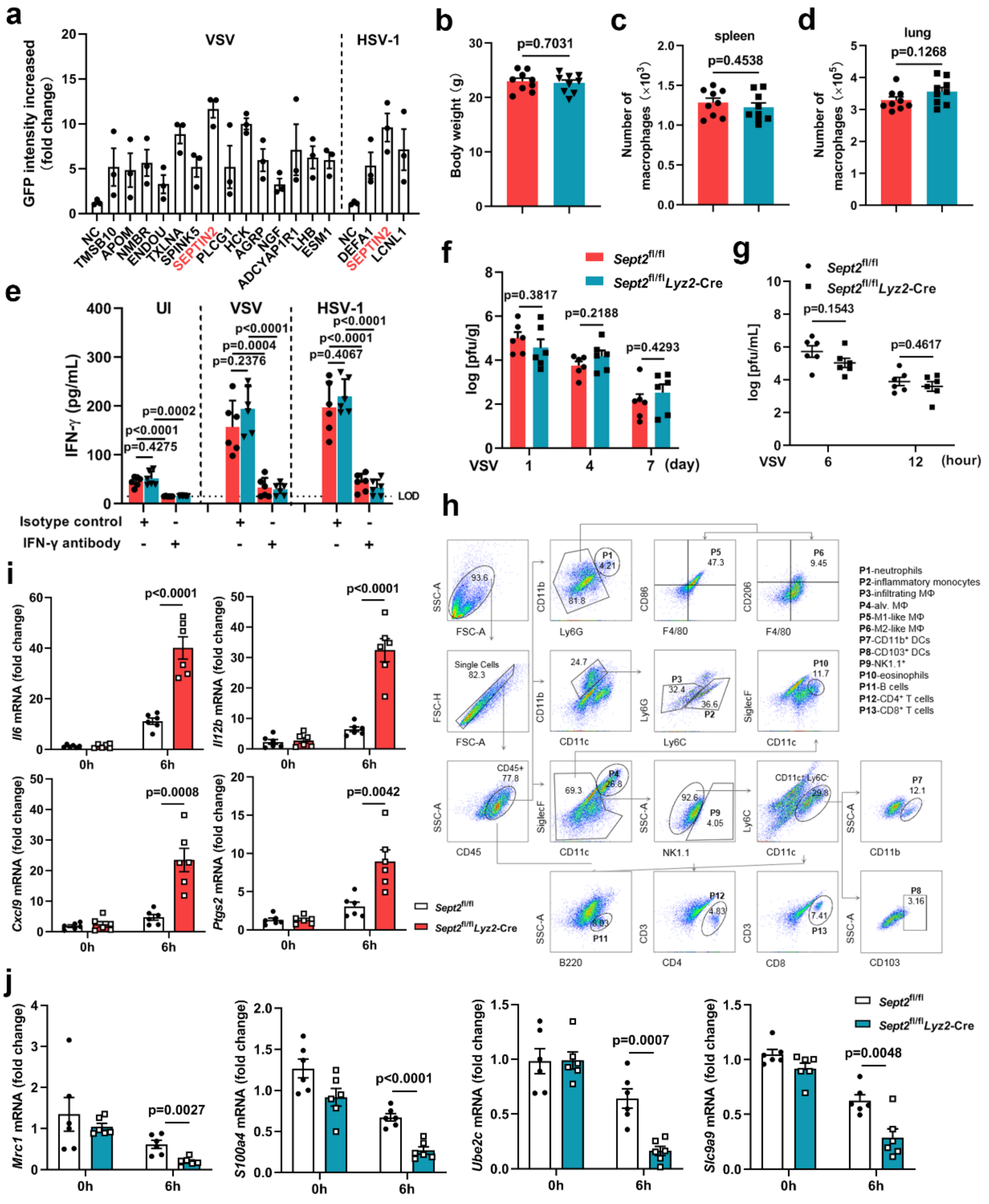

24 Supplementary Figure 1 **SEPT2-deficient mice are phenotypically normal**

a. GFP intensity data obtained from high-content screening were analyzed to investigate regulatory factors in the IFN-γ-independent hyperpolarization. Seventeen genes (14 increased in the VSV infection group and 3 increased in the HSV-1 infection group) are shown. n = 3 in each group (**a**). **b-d.** The body weight (**b**), numbers of macrophages isolated from spleen (**c**) and lung (**d**) of *Sept2*$^{fl/fl}$ *Lyz2*-Cre and *Sept2*$^{fl/fl}$ mice (without infection). n = 9 in each group (**b-d**). **e.** *Sept2*$^{fl/fl}$ *Lyz2*-Cre and *Sept2*$^{fl/fl}$ mice were infected with 1×10^7 PFU VSV or 1×10^8 PFU HSV-1. Daily injection of αIFN-γ (12 mg/kg) or its isotype control from 1 day before infection. The secretion of IFN-γ in BALF at 7 dpi was detected. n = 6 in each group (**e**). LOD: limit of detection. **f.** *Sept2*$^{fl/fl}$ *Lyz2*-Cre and *Sept2*$^{fl/fl}$ mice were infected with 1×10^7 PFU VSV. Viral burdens in lungs at 1, 4 and 7 dpi were detected. **g.** PMs were infected with VSV (MOI = 1) for 6 and 12 hours. The viral titres were measured by plaque assays. n = 6 in each group (**f, g**). **h.** Gating strategy for analysis of innate immune cell populations.**i, j.** qRT-PCR analysis of upregulation markers (*Il6*, *Il12b*, *Cxcl9* and *Ptgs2*) (**i**) and downregulation markers (*Mrc1*, *S100a4*, *Ube2c* and *Slc9a9*) (**j**) of M1-like polarization in *Sept2*$^{fl/fl}$ *Lyz2*-Cre and *Sept2*$^{fl/fl}$ PMs after being infected with VSV (MOI = 1) for 6 hours. The data were normalized to GAPDH expression (**i, j**). n = 6 in each group (**i, j**).

Data are shown as the mean ± s.e.m. (**a-g, i, j**). One-way ANOVA followed by Bonferroni post *hoc* test (**a-g, i, j**) was used for data analysis. Abbreviation: UI, uninfected. Source data are provided as a Source Data file.

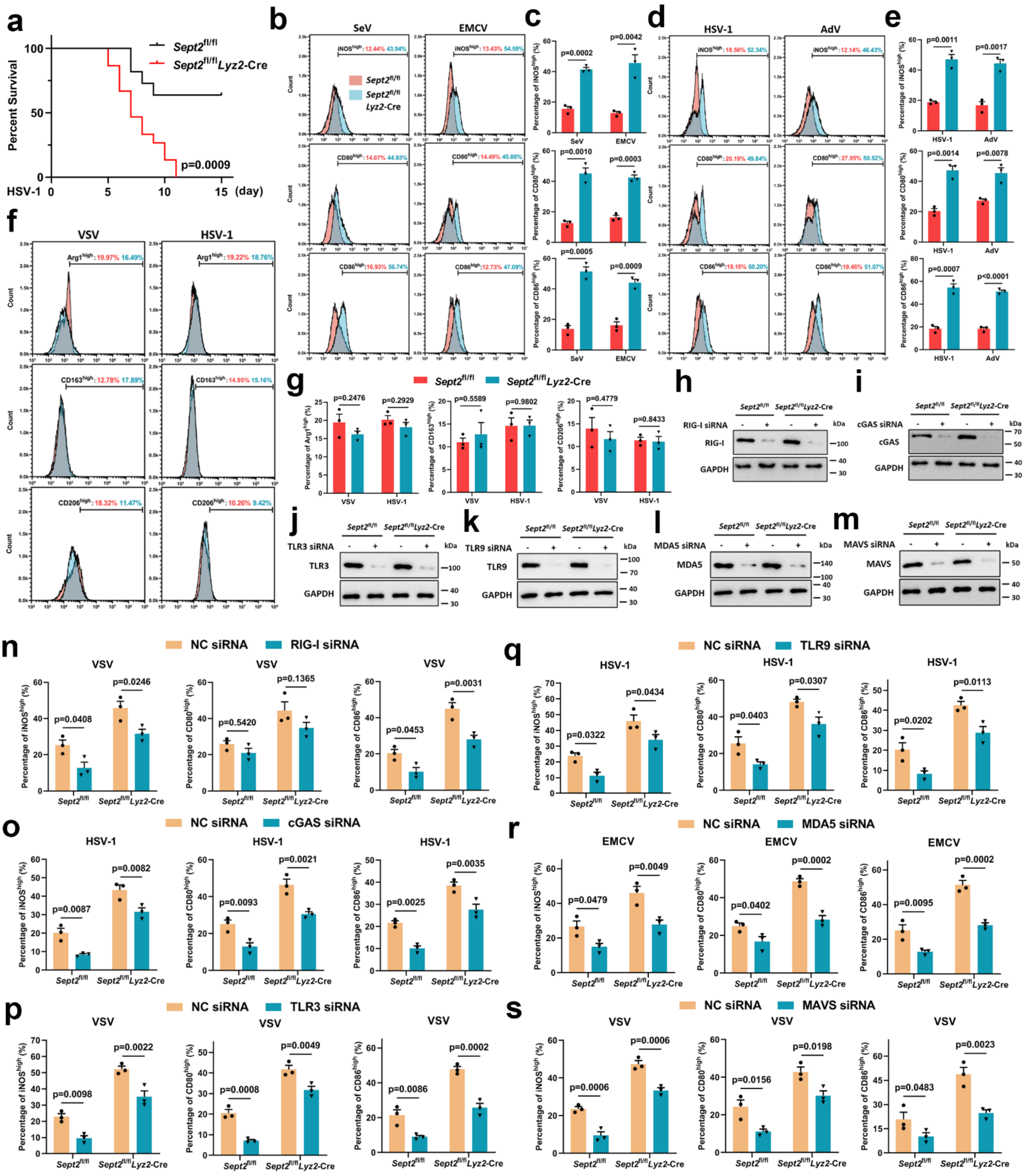

Supplementary Figure 2 **SEPT2-regulated macrophage activation is independent of nucleic acid sensing PRRs**

a. Survival of *Sept2*$^{fl/fl}$ *Lyz2*-Cre (n = 15) and *Sept2*$^{fl/fl}$ (n = 11) mice intraperitoneally infected with 1×10^8 PFU HSV-1. Daily injection of αIFN-γ (12 mg/kg) from 1 day before infection to the end of the experiments was performed to block IFN-γ signaling. **b-e.** Flow cytometry analysis of iNOS, CD80 and CD86 in *Sept2*$^{fl/fl}$ *Lyz2*-Cre and *Sept2*$^{fl/fl}$ PMs after being infected with RNA viruses (SeV [MOI = 1], EMCV [MOI = 1]) (**b**), or DNA viruses (HSV-1 [MOI = 5], Adv [MOI = 1]) (**c**) for 12 hours, respectively. Quantitative data are graphed in (**c, e**). n = 3 in each group (**b-e**). **f, g.** Flow cytometry analysis of Arg-1, CD163 and CD206 in *Sept2*$^{fl/fl}$ *Lyz2*-Cre and *Sept2*$^{fl/fl}$ PMs after being infected with VSV (MOI = 1) or HSV-1 (MOI = 5) for 12 hours (**f**). The gating of Arg-1^{high}, CD163^{high} and CD206^{high} populations was determined against those of the uninfected control. Quantitative data are graphed in (**g**). n = 3 in each group (**f, g**). **h-m.** The knockdown efficiency of RNA interference was detected by western blotting. **n-s.** Quantitative flow cytometry data of iNOS, CD80 and CD86 in *Sept2*$^{fl/fl}$ *Lyz2*-Cre and *Sept2*$^{fl/fl}$ PMs after transfection with the indicated siRNAs 12 hours after VSV (MOI =1, **n, p, s**), HSV-1 (MOI = 5, **o, q**) or EMCV (MOI = 1, **r**) infection. n = 3 in each group (**h-s**).

Data are shown as Kaplan-Meier curves (**a**) or the mean ± s.e.m. (**c, e, g, n-s**). Log-rank (Mantel-Cox) test (**a**) or one-way ANOVA followed by Bonferroni post *hoc* test (**c, e, g, n-s**) were used for data analysis. Abbreviation: NC, negative control. Source data are provided as a Source Data file.

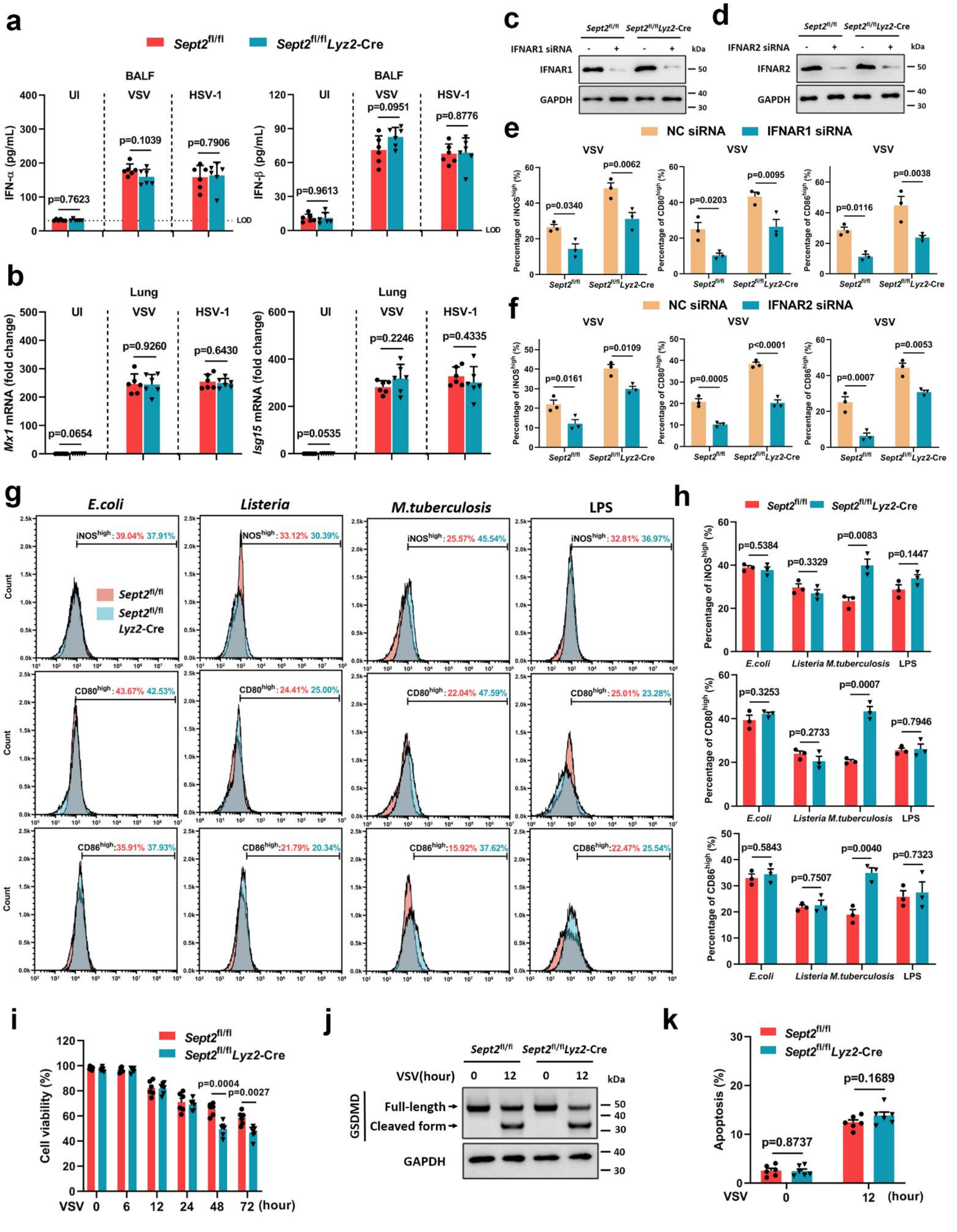

Supplementary Figure 3 **SEPT2 plays different roles in viral and bacterial**

infections

a, b. *Sept2*$^{fl/fl}$ *Lyz2*-Cre and *Sept2*$^{fl/fl}$ mice were infected with 1×10^7 PFU VSV or 1×10^8 PFU HSV-1. The secretion of IFN-α, IFN-β in BALF at 7 dpi (**a**) and the mRNA expression of *Mx1* and *Isg15* in lung tissues at 7 dpi (**b**) were detected. The data were normalized to GAPDH expression (**b**). n = 6 in each group (**a, b**). LOD: limit of detection. **c, d.** The knockdown efficiency of IFNAR1 and IFNAR2 siRNAs was examined by western blotting. **e, f**. Quantitative flow cytometry data of iNOS, CD80 and CD86 in *Sept2*$^{fl/fl}$ *Lyz2*-Cre and *Sept2*$^{fl/fl}$ PMs after transfection with the indicated siRNAs 12 hours after VSV infection (**e, f**). n = 3 in each group (**c-f**). **g, h.** Flow cytometry analysis of iNOS, CD80 and CD86 in *Sept2*$^{fl/fl}$ *Lyz2*-Cre and *Sept2*$^{fl/fl}$ PMs after being infected with *E.coil* (MOI = 20), *Listeria* (MOI = 20), *M.tuberculosis* (MOI = 10) or stimulated with LPS (100 ng/mL) for 12 hours (**g**). The gating of iNOS^{high}, CD80^{high} and CD86^{high} populations was determined against those of the uninfected control. Quantitative data are graphed in (**h**). n = 3 in each group (**g, h**). **i.** Cell viability of *Sept2*$^{fl/fl}$ *Lyz2*-Cre and *Sept2*$^{fl/fl}$ PMs after being infected with VSV (MOI = 1) for the indicated times. n = 6 in each group (**i**). **j, k.** PMs were infected with VSV (MOI = 1) for 12 hours. The expression of full-length GSDMD and its cleaved form was detected by western blotting (**j**). n = 3 in each group (**j**). The apoptosis was detected by flow cytometry (**k**). n = 6 in each group (**k**).

Data are shown as the mean ± s.e.m. (**a, b, e, f, h, i, k**). One-way ANOVA followed by Bonferroni post *hoc* test (**a, b, e, f, h, i, k**) was used for data analysis. Abbreviation: NC, negative control. Source data are provided as a Source Data file.

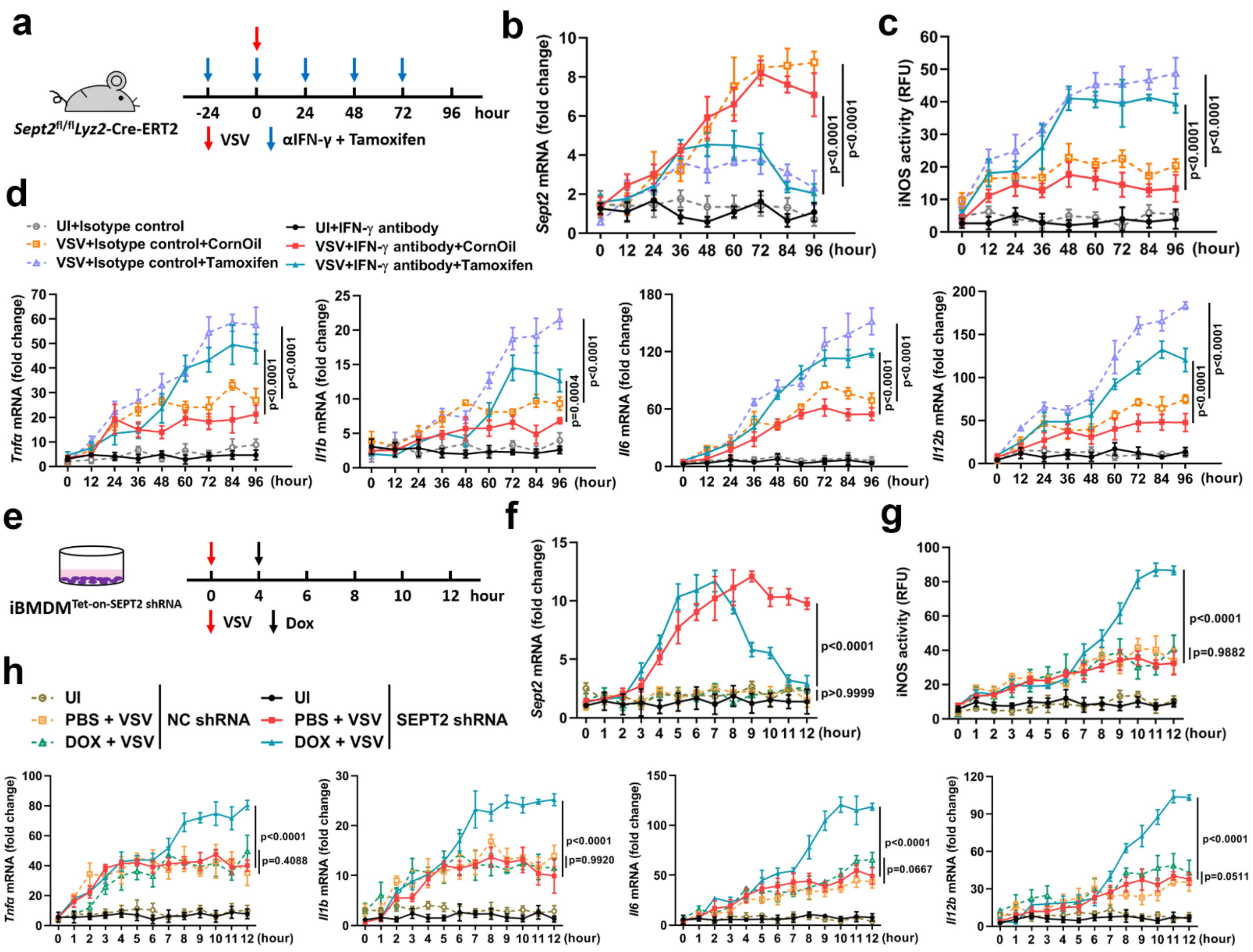

Supplementary Figure 4 **SEPT2 controls IFN-γ-independent hyperpolarization**

a. *Sept2*$^{fl/fl}$ *Lyz2*-Cre-ERT2 mice were intraperitoneally injected with Tamoxifen (50 mg/kg) every 24 hours for 5 consecutive days. After the second dose of tamoxifen, mice were intraperitoneally infected with 1×10^7 PFU VSV. Daily intraperitoneal injection of αIFN-γ (12 mg/kg) was performed to block IFN-γ signaling in all groups. **b-d.** Relative expression of SEPT2 (**b**), iNOS activity (**c**) and proinflammatory cytokines (*Tnfα*, *Il1b*, *Il6* and *Il12b*) (**d**) in mice PMs were detected at indicated time points. The qRT-PCR data were normalized to GAPDH expression (**b, d**). n = 3 in each group (**b-d**). **e.** Schematic diagram of DOX (1 μg/mL)-induced SEPT2-

knockdown in pLKO-Tet-on-SEPT2 shRNA stably transfected iBMDMs after VSV infection (MOI = 1). **f-h.** Relative expression of SEPT2 (**f**), iNOS activity (**g**) and proinflammatory cytokines levels (*Tnfα*, *Il1b*, *Il6* and *Il12b*) (**h**) were detected at indicated time points. The qRT-PCR data were normalized to GAPDH expression (**f, h**). n = 3 in each group (**f-h**).

Data are shown as the mean ± s.e.m. (**b-d, f-h**). One-way ANOVA followed by Bonferroni post *hoc* test (**b-d, f-h**) was used for data analysis. Abbreviation: UI, uninfected. NC, negative control. Source data are provided as a Source Data file.

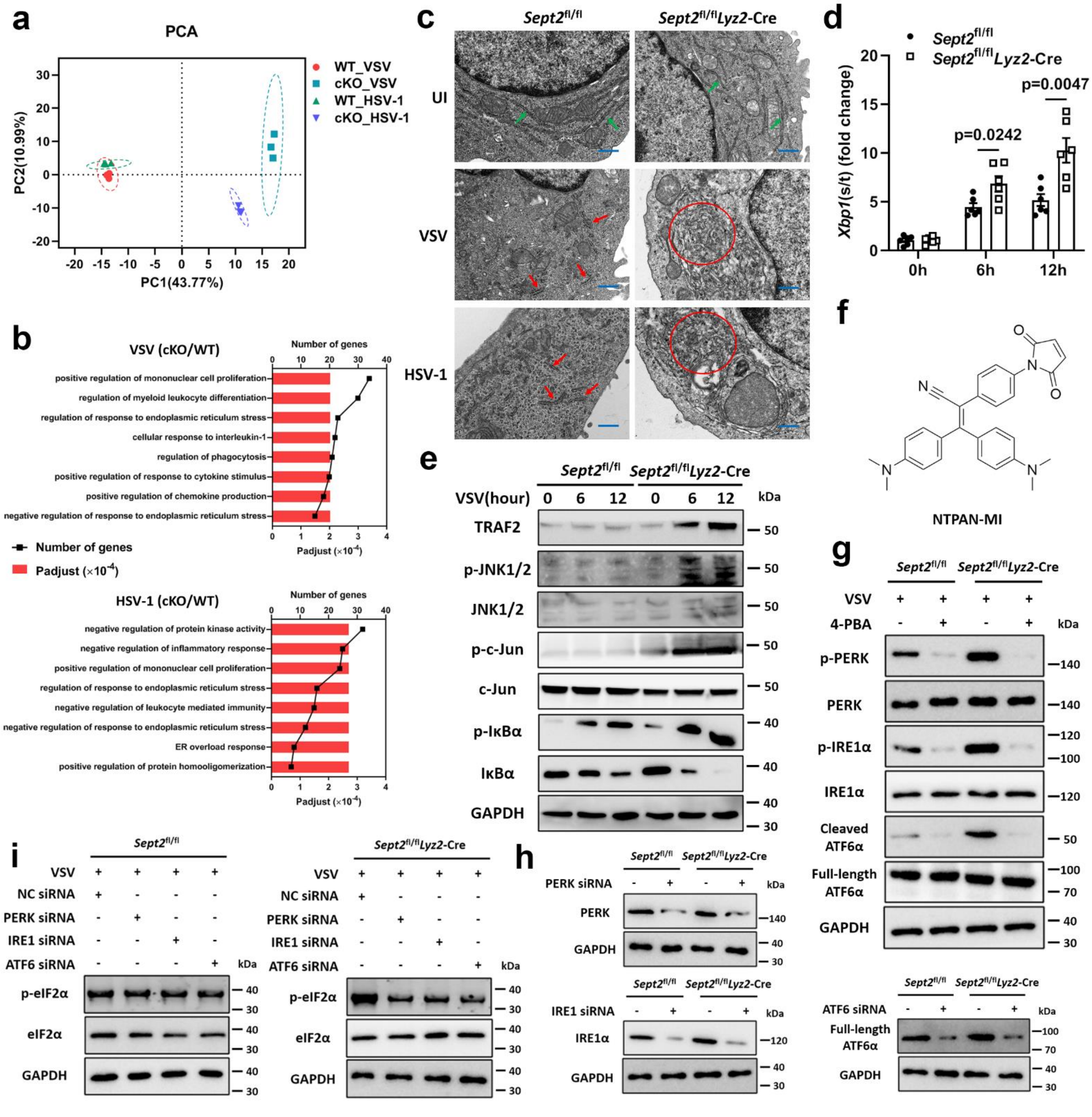

Supplementary Figure 5 **SEPT2-regulated polarization is associated with ER stress**

a. The consistency of each sample submitted for RNA-sequencing was analyzed by PCA. **b.** *Sept2*$^{fl/fl}$ *Lyz2*-Cre and *Sept2*$^{fl/fl}$ PMs were infected with VSV (MOI = 1) or HSV-1 (MOI = 5) for 12 hours, followed by total RNA extraction and RNA-

sequencing. GO analysis was used to determine the enrichment of pathways. **c.** Transmission electron microscopy of *Sept2*$^{fl/fl}$ *Lyz2*-Cre and *Sept2*$^{fl/fl}$ PMs after being infected with VSV (MOI = 1) or HSV-1 (MOI = 5) for 12 hours. Green arrow: normal ER. Red arrow and circle: swollen ER and degranulated ribosomes. Scale bar = 5 μm. n = 3 in each group (**c**). **d, e.** *Sept2*$^{fl/fl}$ *Lyz2*-Cre and *Sept2*$^{fl/fl}$ PMs were infected with VSV (MOI = 1). The XBP1 mRNA splicing levels were detected by qRT-PCR (**d**), and activation of M1-like polarization related signaling pathways were detected by immunoblots (**e**) at 6 and 12 hours post infection. n = 6 (**d**) or n = 3 (**e**) in each group. **f.** The chemical structural of NTPAN-MI probe. **g.** *Sept2*$^{fl/fl}$ and *Sept2*$^{fl/fl}$ *Lyz2*-Cre PMs were infected with VSV (MOI = 1) in the absence or presence of 4-PBA (5 mM) for 12 hours. The activation of PERK, IRE1 and ATF6 pathways was detected by western blotting. n = 3 in each group (**g**). **h**. The knockdown efficiency of PERK, IRE1 and ATF6 siRNAs. **i**. *Sept2*$^{fl/fl}$ and *Sept2*$^{fl/fl}$ *Lyz2*-Cre PMs were transfected with PERK, IRE1 or ATF6 siRNA for 24 hours, followed by infection of VSV (MOI = 1) for 12 hours. The activation of eIF2α pathway was detected by western blotting. n = 3 in each group (**h, i**).

Data are shown as the mean ± s.e.m. (**d**). One-way ANOVA followed by Bonferroni post *hoc* test (**d**) was used for data analysis. Abbreviation: UI, uninfected. NC, negative control. Source data are provided as a Source Data file.

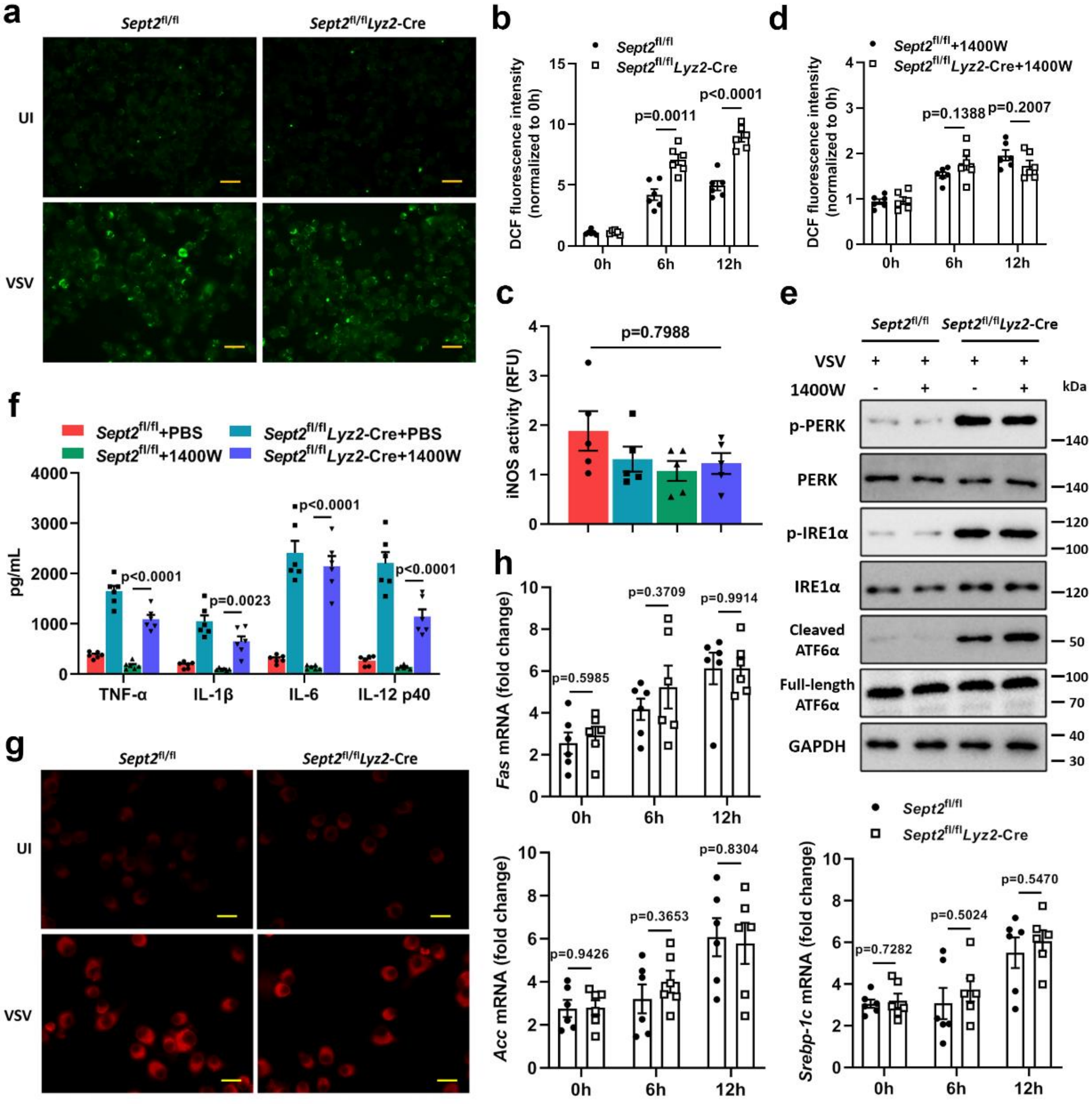

Supplementary Figure 6 **SEPT2 deficiency-associated ER stress is induced by the accumulation of unfolded proteins**

a. The Ca^{2+} concentrations are similar in VSV-infected WT and SEPT2-deficient cells. *Sept2*$^{fl/fl}$ *Lyz2*-Cre and *Sept2*$^{fl/fl}$ PMs were infected with VSV (MOI = 1) for 12 hours. The concentration of Ca^{2+} in ER was detected by Mag-Fluo4 probe. Scale bar =

50 μm. n = 3 in each group (**a**). **b-f.** *Sept2*$^{fl/fl}$ *Lyz2*-Cre and *Sept2*$^{fl/fl}$ PMs were infected with VSV (MOI = 1) for 12 hours. **b.** The intracellular ROS levels were detected by H_2DCFDA probe. **c-f.** 1400W (100 μM) was used to treat cells during VSV infection. The inhibitory effect of 1400W on iNOS activity was shown in (**c**). The intracellular ROS (**d**), UPR levels (**e**) and proinflammatory cytokines (**f**) under 1400W treatment were detected by H_2DCFDA probe, western blotting and ELISA, respectively. n = 6 (**b-d, f**) or n = 3 (**e**) in each group. **g, h.** *Sept2*$^{fl/fl}$ *Lyz2*-Cre and *Sept2*$^{fl/fl}$ PMs were infected with VSV for 12 hours. **g.** Intracellular lipid content was detected by BODIPY 647 staining. Scale bar = 50 μm. **h.** The expression of lipid metabolism-related genes (*Fas, Acc* and *Srebp-1c*) was detected by qRT-PCR. The data were normalized to GAPDH expression (**h**). n = 3 (**g**) or n = 6 (**h**) in each group.
Data are shown as the mean ± s.e.m. (**b-d, f, h**). One-way ANOVA followed by Bonferroni post *hoc* test (**b-d, f, h**) was used for data analysis. Abbreviation: UI, uninfected. Source data are provided as a Source Data file.

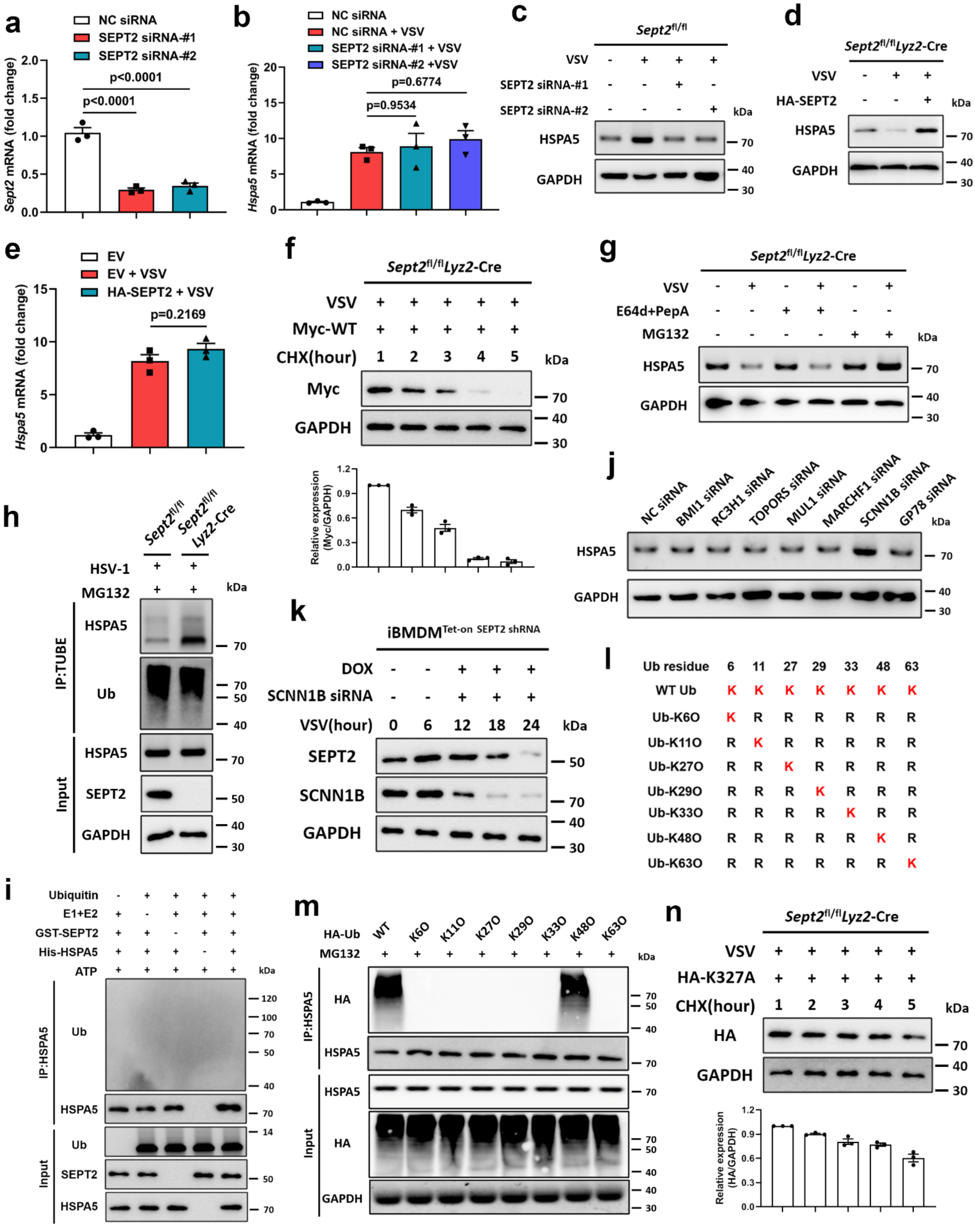

Supplementary Figure 7 **SEPT2 depletion results in the ubiquitination of HSPA5**

by SCNN1B

a. The knockdown efficiency of SEPT2 siRNAs in *Sept2*$^{\mathrm{fl/fl}}$ PMs. **b, c.** qRT-PCR (**b**) and immunoblot analysis (**c**) of HSPA5 in *Sept2*$^{\mathrm{fl/fl}}$ PMs after being transfected with SEPT2 siRNAs and infected with VSV (MOI = 1) for 12 hours. **d, e.** Immunoblot analysis (**d**) and qRT-PCR (**e**) of HSPA5 in *Sept2*$^{\mathrm{fl/fl}}$ *Lyz2*-Cre PMs after being transfected with HA-tagged SEPT2 and infected with VSV (MOI = 1) for 12 hours. n = 3 in each group (**a-e**). **f.** PMs obtained from *Sept2*$^{\mathrm{fl/fl}}$ *Lyz2*-Cre mice were transfected with Myc-tagged WT HSPA5 plasmids and infected with VSV (MOI = 1). CHX (50 μg/mL) was used to inhibit the protein synthesis. The expression of HSPA5 was detected by western blotting. **g.** Immunoblot analysis of HSPA5 in PMs after being pretreated with E64d (10 μg/mL) + PepA (10 μg/mL) or MG132 (10 μg/mL) for 4 hours, followed by VSV infection (MOI = 1) for another 12 hours. **h.** The ubiquitination level of HSPA5 in PMs after being infected with HSV-1 (MOI = 5) for 12 hours. **i.** The ubiquitination of HSPA5 in an *in vitro* ubiquitination system was analyzed by immunoblots. **j.** Screening of potential ubiquitin E3 ligases by siRNAs in *Sept2*$^{\mathrm{fl/fl}}$ *Lyz2*-Cre PMs after being infected with VSV (MOI = 1) for 12 hours. The expression of HSPA5 was analyzed. **k.** The expression of SEPT2 and SCNN1B in Fig. **3h** was detected by western blotting. **l.** Schematic of WT and mutated ubiquitins (K, Lysine; R, Arginine). **m.** The ubiquitination level of HSPA5 in *Sept2*$^{\mathrm{fl/fl}}$ *Lyz2*-Cre PMs after being transfected with HA-tagged WT or mutated ubiquitin and infected with VSV (MOI = 1) for 12 hours. n = 3 in each group (**f-k, m**). **n.** PMs obtained from *Sept2*$^{\mathrm{fl/fl}}$ *Lyz2*-Cre mice were transfected with HA-tagged HSPA5 K327A plasmids

and infected with VSV (MOI = 1). CHX (50 μg/mL) was used to inhibit the protein synthesis. The expression of HSPA5 K327A was detected by western blotting. n = 3 in each group (**n**).

MG132 (10 μg/mL) was used to inhibit the degradation of HSPA5 (**g, m**). Data are shown as the mean ± s.e.m. (**a, b, e, f, n**). One-way ANOVA followed by Bonferroni post *hoc* test (**a, b, e**) was used for data analysis. Abbreviation: UI, uninfected. NC, negative control. EV, empty vector. Source data are provided as a Source Data file.

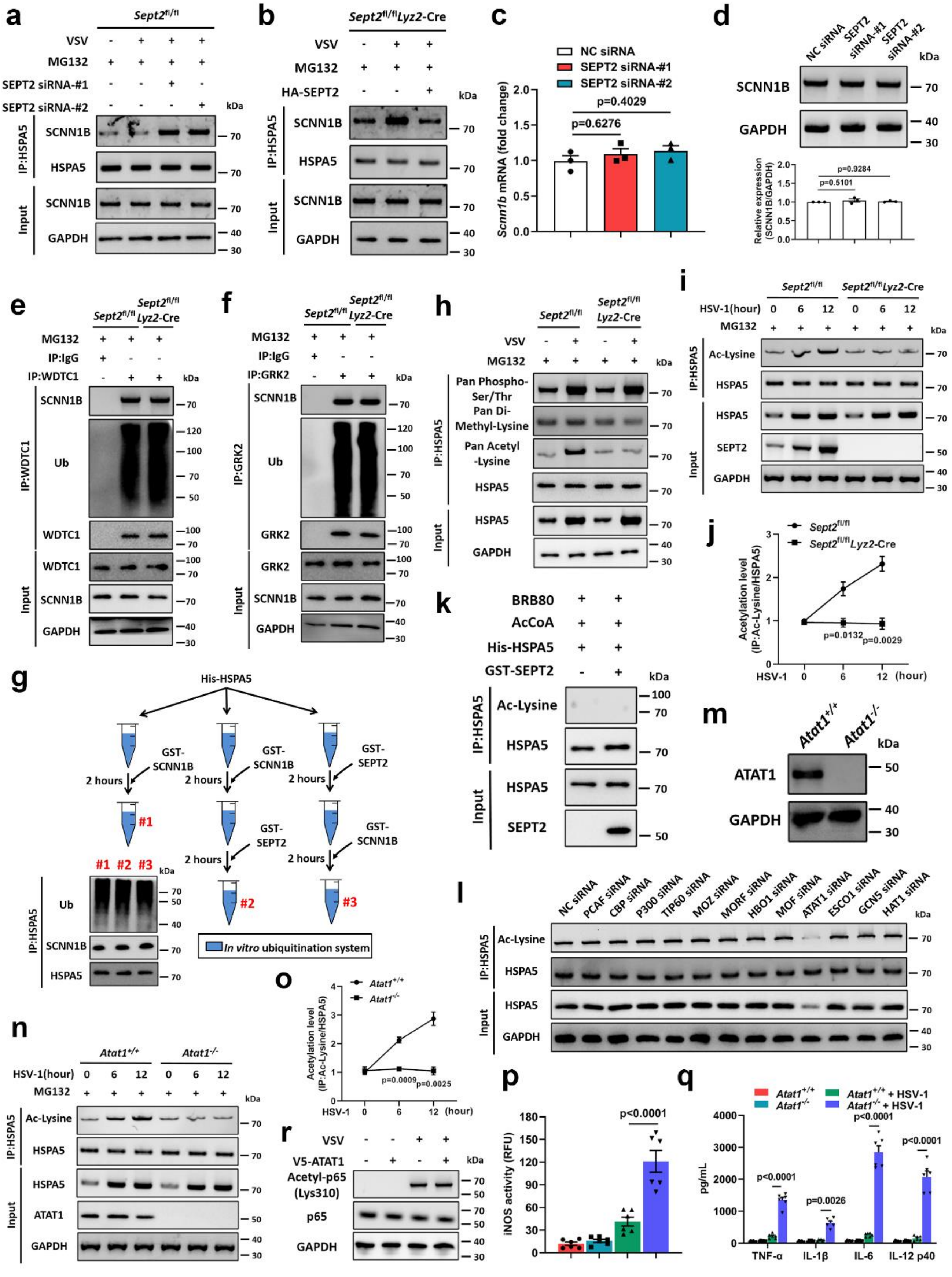

223 Supplementary Figure 8 **ATAT1 is required for SEPT2-regulated HSPA5**

acetylation

a, b. Immunoprecipitation analysis of the interaction between HSPA5 and SCNN1B in PMs after being transfected with SEPT2 siRNAs (**a**) or HA-tagged SEPT2 (**b**), and infected with VSV (MOI = 1) for 12 hours. **c, d.** qRT-PCR (**c**) and immunoblot analysis (**d**) of SCNN1B in *Sept2*$^{fl/fl}$ PMs after being transfected with SEPT2 siRNAs and then infected with VSV (MOI = 1) for 12 hours. Quantitative data are graphed below the immunoblots (**d**). **e, f.** The ubiquitination levels of WDTC1 (**e**) and GRK2 (**f**) were detected. **g.** Schematic diagram of an *in vitro* ubiquitination system. The interaction of HSPA5 and SCNN1B, and the ubiquitination of HSPA5 were detected by immunoprecipitation. n = 3 in each group (**a-g**). **h.** The modifications of HSPA5 in PMs were detected by immunoprecipitation analysis. **i, j.** Immunoprecipitation analysis of acetylated HSPA5 in PMs after being infected with HSV-1 (MOI = 5) (**i**). Quantitative data are graphed in (**j**). **k.** The acetylation of HSPA5 modified by SEPT2 was identified using an *in vitro* acetylation system described in Fig. **4f**. n = 3 in each group (**h-k**). **l.** Screening of potential acetylase by siRNAs in iBMDMs after being infected with VSV (MOI = 1) for 12 hours. The acetylation of HSPA5 was analyzed. **m.** Validation of *Atat1*$^{+/+}$ and *Atat1*$^{-/-}$ iBMDMs. **n, o.** Immunoprecipitation analysis of acetylated HSPA5 in iBMDMs after being infected with HSV-1 (MOI = 5) (**n**). Quantitative data are graphed in (**o**). n = 3 in each group (**l-o**). **p, q.** iNOS activities (**p**) and secretion of proinflammatory cytokines (**q**) in iBMDMs after being infected with HSV-1 (MOI = 5). n = 6 in each group (**p, q**). **r.** WT iBMDMs were transfected with V5-tagged ATAT1 for 24 hours, followed by VSV infection (MOI = 1) for 12

hours. The acetylation of p65 was detected. n = 3 in each group (**r**).

MG132 (10 μg/mL) was used to inhibit the protein degradation (**a, b, e, f, h, i, j, n, o**).

Data are shown as the mean ± s.e.m. (**c, d, j, o-q**). One-way ANOVA followed by Bonferroni post *hoc* test (**c, d, j, o-q**) was used for data analysis. Abbreviation: NC, negative control. Source data are provided as a Source Data file.

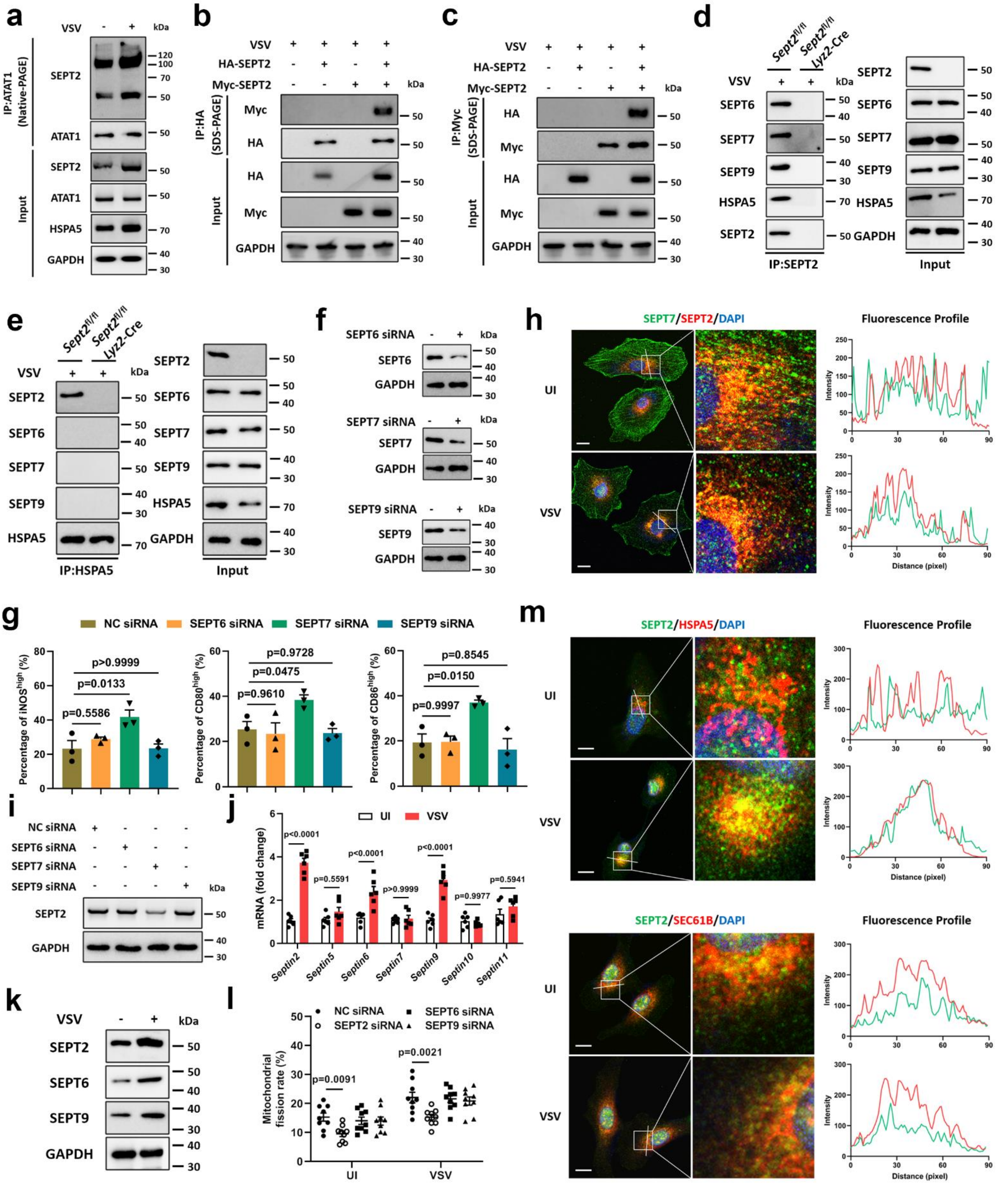

268 Supplementary Figure 9 **SEPT2 regulates macrophage activation independently of**

269 **other SEPTINs**

a. Immunoprecipitation analysis of the interaction of ATAT1 and SEPT2 in iBMDMs after being infected with VSV (MOI = 1) for 12 hours. **b, c.** Immunoprecipitation analysis of SEPT2 oligomerization in iBMDMs after being transfected with HA-tagged SEPT2 or Myc-tagged SEPT2. n = 3 in each group (**a-c**). **d, e.** Immunoprecipitation analysis of the interaction between SEPT2-HSPA5 complex and other SEPTINs in PMs after being infected with VSV (MOI = 1) for 12 hours. n = 3 in each group (**d, e**). **f.** The knockdown efficiency of SEPT6, SEPT7 and SEPT9. **g.** Quantitative flow cytometry data of iNOS, CD80 and CD86 in iBMDMs after being transfected with the indicated siRNAs for 24 hours and infected with VSV (MOI = 1) for 12 hours. **h.** iBMDMs were infected with VSV (MOI =1) for 12 hours. The co-localization of SEPT2 and SEPT7 was analyzed. Scale bar = 5 μm. **i.** iBMDMs were transfected with SEPT6, SEPT7 or SEPT9 siRNA for 24 hours, followed by infection of VSV (MOI = 1) for 12 hours. The expression of SEPT2 was detected. n = 3 in each group (**f-i**). **j, k.** iBMDMs were infected with VSV (MOI = 1) for 12 hours. The mRNA and protein expressions of SEPTINs were detected. **l.** iBMDMs were transfected with SEPT2, SEPT6 or SEPT9 siRNA for 24 hours, followed by VSV infection (MOI = 1) for 12 hours. The mitochondrial fission rates were detected by live cell imaging. n = 6 (**j**) or n = 3 (**k**) or n = 9 (**l**) in each group. **m.** iBMDMs were infected with VSV (MOI =1) for 12 hours. The co-localization of SEPT2 with HSPA5 and SEC61B was analyzed. Scale bar = 5 μm. n = 3 in each group (**m**).

Data are shown as the mean ± s.e.m. (**g, j, l**). One-way ANOVA followed by Bonferroni post *hoc* test (**g, j, l**) was used for data analysis. Abbreviation: UI,

uninfected. NC, negative control. Source data are provided as a Source Data file.

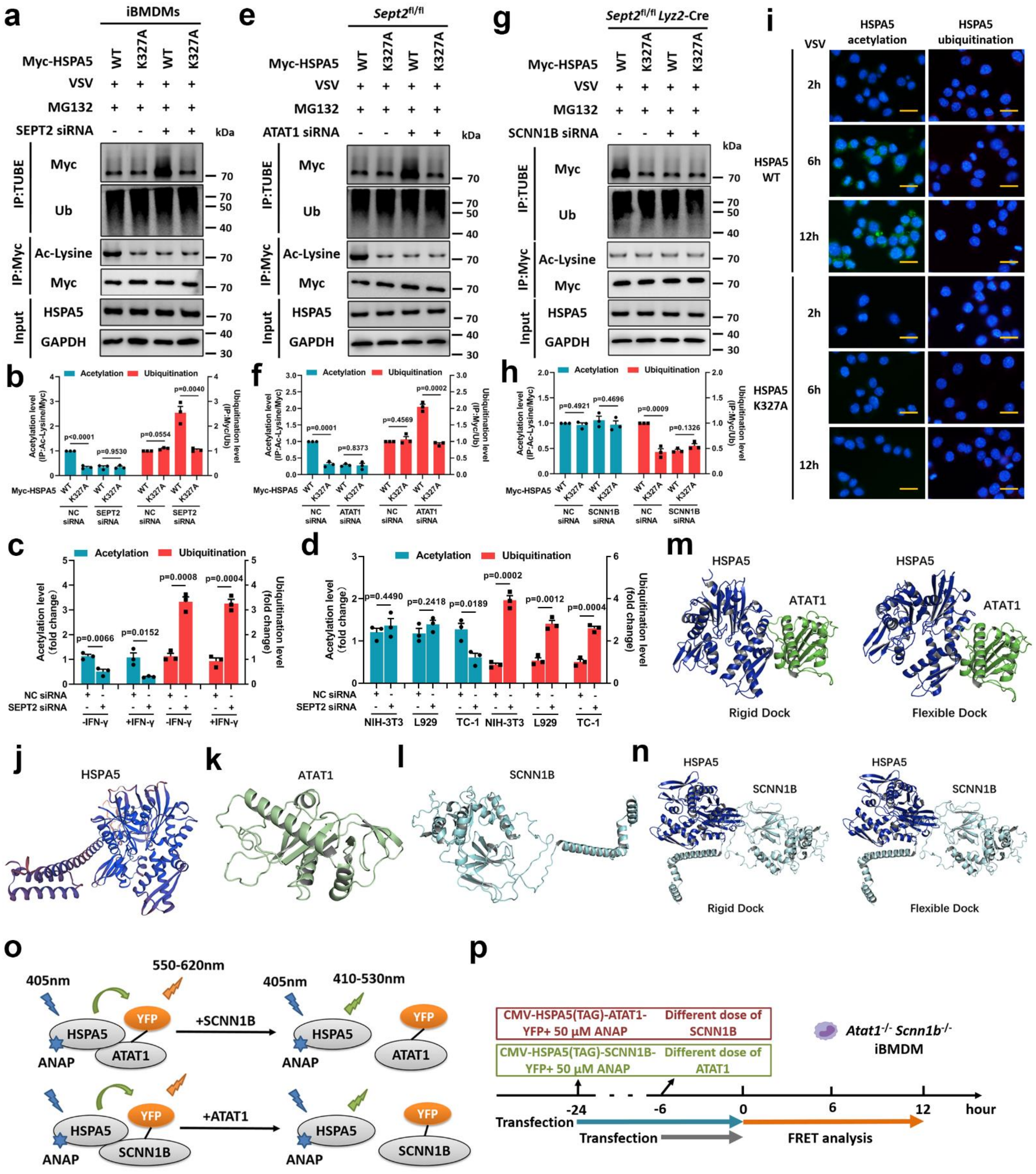

314 Supplementary Figure 10 **HSPA5 K327 is a common site for acetylation and**

315 **ubiquitination**

a, b. The acetylation and ubiquitination levels of HSPA5 in iBMDMs after being transfected with Myc-tagged HSPA5, HSPA5 K327A, or SEPT2 siRNA and infected with VSV (MOI = 1) for 12 hours (**a**). Quantitative data are graphed in (**b**). n = 3 in each group (**a, b**). **c.** Quantitative data of the acetylation and ubiquitination levels of HSPA5 in iBMDMs after being stimulated with IFN-γ (50 ng/mL) and infected with VSV (MOI = 1) for 12 hours. n = 3 in each group (**c**). **d.** Quantitative data of the acetylation and ubiquitination levels of HSPA5 in NIH-3T3, L929 and TC-1 cells after being transfected with SEPT2 siRNA and infected with VSV (MOI = 1) for 12 hours. n = 3 in each group (**d**). **e. f.** The acetylation and ubiquitination levels of HSPA5 in PMs after being transfected with Myc-tagged HSPA5, HSPA5 K327A, or ATAT1 siRNA and infected with VSV (MOI = 1) for 12 hours (**e**). Quantitative data are graphed in (**f**). **g, h.** The acetylation and ubiquitination levels of HSPA5 in PMs after being transfected with Myc-tagged HSPA5, HSPA5 K327A, or SCNN1B siRNA and infected with VSV (MOI = 1) for 12 hours (**g**). Quantitative data are graphed in (**h**). **i.** PLA of the acetylation and ubiquitination status of HSPA5 in iBMDMs after being transfected with Myc-tagged HSPA5 or HSPA5 K327A and infected with VSV (MOI = 1). Scale bar = 20 μm. n = 3 in each group (**e-i**). **j-l.** Overall structure of the inactive structure-removed HSPA5 (**j**), ATAT1 (**k**) and SCNN1B (**l**). **m, n.** The rigid dock and flexible dock structures of ATAT1-HSPA5 (**m**) and SCNN1B-HSPA5 (**n**) complexes. **o, p.** Schematic of FRET analysis.

MG132 (10 μg/mL) was used to inhibit the proteasomal degradation of HSPA5 (**a-i**). Data are shown as the mean ± s.e.m. (**b-d, f, h**). One-way ANOVA followed by

Bonferroni post *hoc* test (**c, d**) was used for data analysis. Abbreviation: NC, negative control. Source data are provided as a Source Data file.

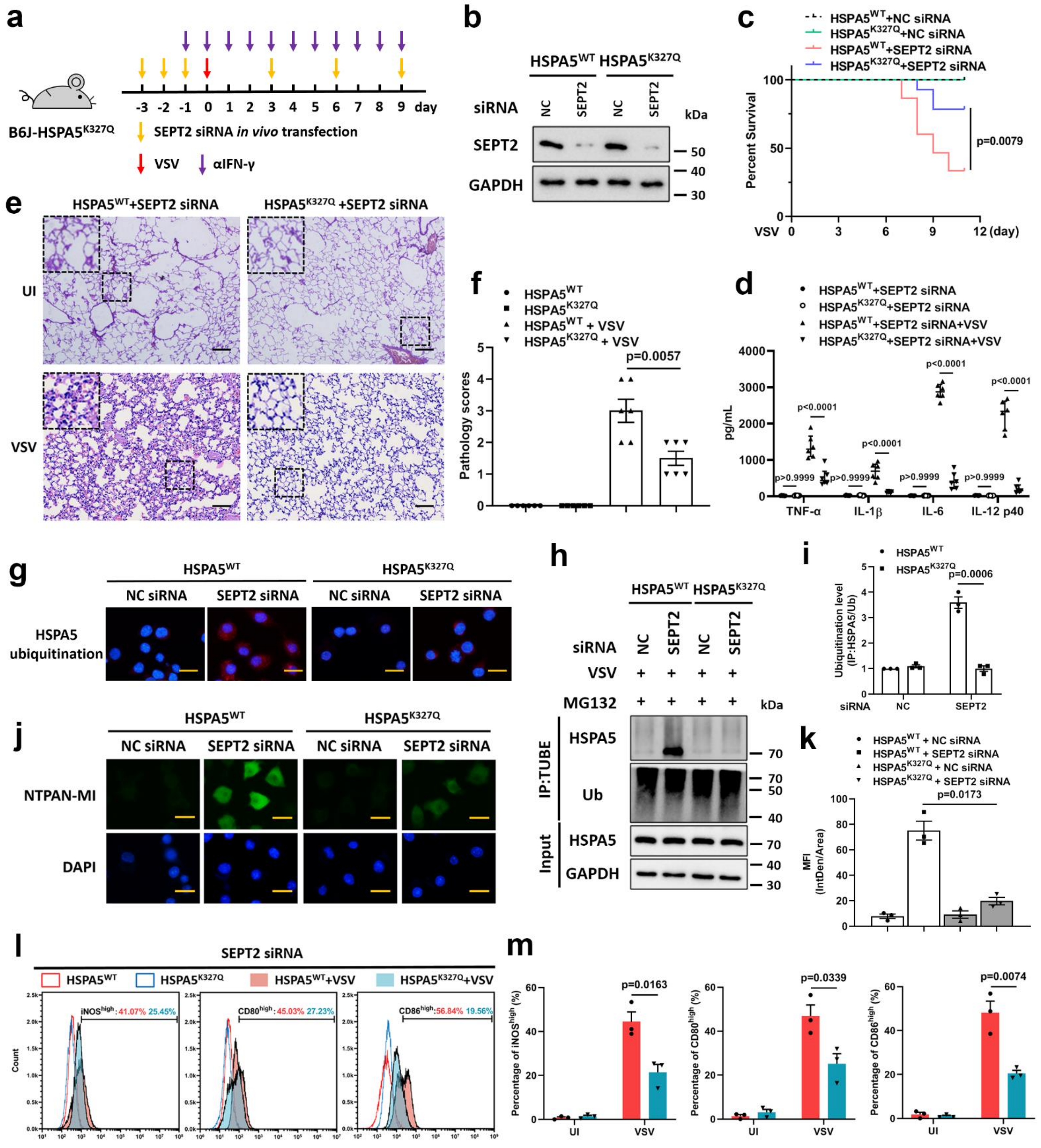

360 Supplementary Figure 11 **HSPA5^{K327Q} mice resist hyperinflammation caused by**

361 **SEPT2 knockdown**

362 **a.** Schematic diagram of SEPT2 siRNA *in vivo* transfection in B6/JGpt-

Hspa5$^{em1(K327Q)}$ background. Daily injection of αIFN-γ (12 mg/kg) from 1 day before infection to the end of the experiments was performed to block IFN-γ signaling. **b.** The *in vivo* transfection efficiency of SEPT2 siRNA was detected in lungs at 7 dpi. n = 3 in each group (**b**). **c.** Survival of B6J-HSPA5^{WT} (NC siRNA, n = 14. SEPT2 siRNA, n = 15) and B6J-HSPA5^{K327Q} (NC siRNA, n = 16. SEPT2 siRNA, n = 14) mice intraperitoneally infected with 1×10^7 PFU VSV. **d.** ELISA analysis of proinflammatory cytokines in mice BALF at 7 dpi. n = 6 in each group (**d**). **e, f.** H&E staining (**e**) and the pathology scores (**f**) of lung lesions in mice at 7 dpi. Scale bar = 400 μm. n = 6 in each group (**e, f**). **g-m.** B6J-HSPA5^{WT} and B6J-HSPA5^{K327Q} mice PMs were transfected with NC siRNA or SEPT2 siRNA and then infected with VSV (MOI = 1) for 12 hours. **g-i.** The ubiquitination level of HSPA5 was detected by PLA (**g**) and TUBE analysis (**h**). Scale bar = 20 μm (**g**). Quantitative data of the TUBE analysis are graphed in (**i**). **j, k.** NTPAN-MI probe was used to determine the accumulation of unfolded proteins (**j**). Scale bar = 20 μm. The MFI was quantitated and shown as IntDen/Area (**k**). **l, m.** The expression of iNOS, CD80 and CD86 was detected by flow cytometry (**l**). The gating of iNOS^{high}, CD80^{high} and CD86^{high} populations was determined against those of the uninfected control (the blank peaks). Quantitative data are graphed in (**m**). n = 3 in each group (**g-m**). MG132 (10 μg/mL) was used to inhibit the proteasomal degradation of HSPA5 (**g-i**). Data are shown as Kaplan-Meier curves (**c**) and the mean ± s.e.m. (**d, f, i, k, m**). Log-rank (Mantel-Cox) test (**c**) and one-way ANOVA followed by Bonferroni post *hoc* test (**d, f, i, k, m**) was used for data analysis. Abbreviation: NC, negative control. Source

data are provided as a Source Data file.

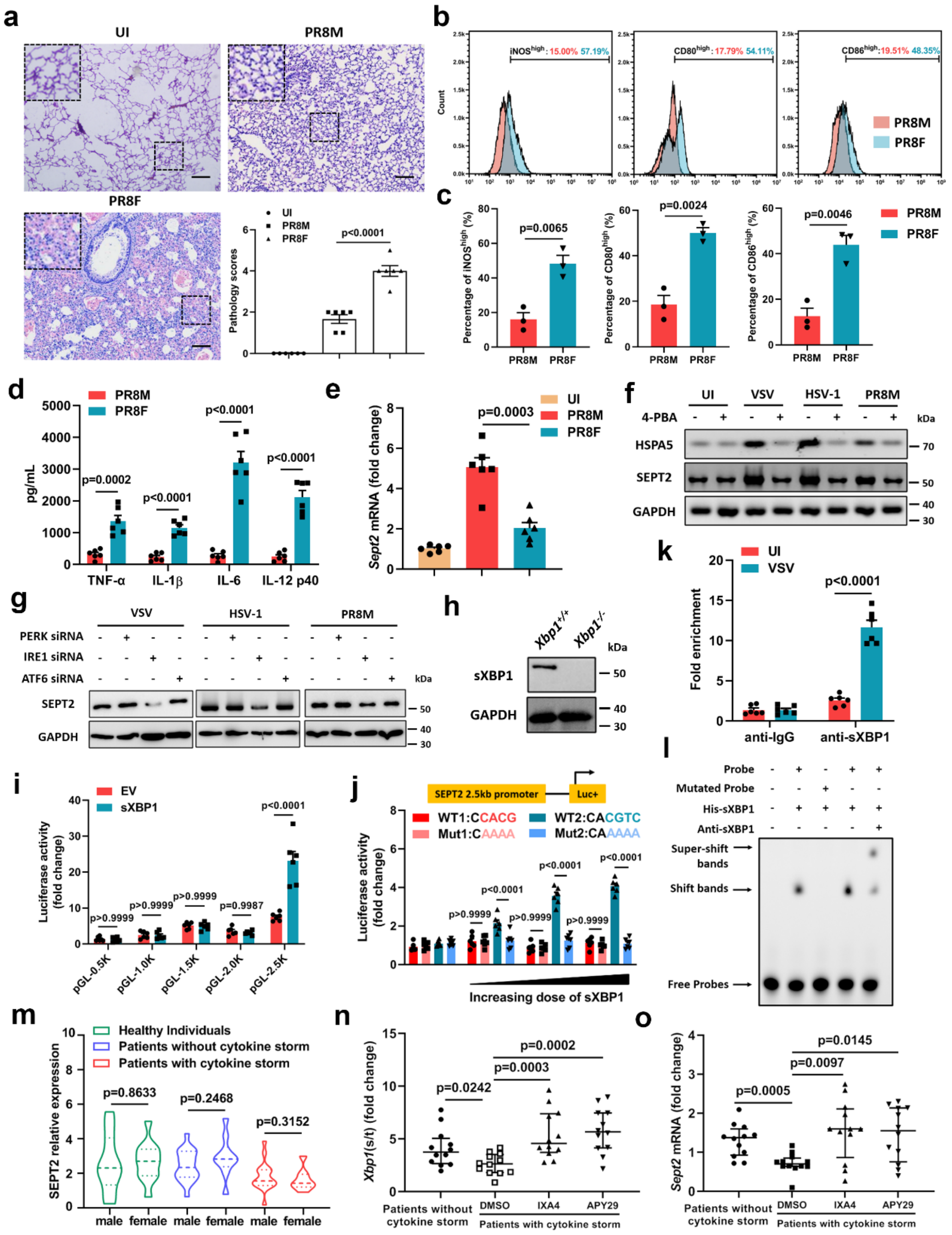

407 Supplementary Figure 12 **XBP1 is a transcription factor of SEPT2**

a. H&E staining of lung lesions in C57BL/6J mice intraperitoneally infected with 1×10^4 PFU PR8M or PR8F at 7 dpi. Daily intraperitoneal injection of αIFN-γ (12 mg/kg) was performed to block IFN-γ signaling. The pathology scores were quantitated and shown as histogram. Scale bar = 400 μm. n = 6 in each group (**a**). **b, c.** Flow cytometry analysis of iNOS, CD80 and CD86 in C57BL/6J mice PMs after being infected with PR8M or PR8F (MOI = 1) for 12 hours (**b**). Quantitative data are graphed in (**c**). n = 3 in each group (**b, c**). **d.** The secretion of proinflammatory cytokines was detected by ELISA. n = 6 in each group (**d**). **e.** C57BL/6J mice PMs were infected with PR8M or PR8F (MOI = 1) for 12 hours. The expression of SEPT2 was detected. n = 6 in each group (**e**). **f.** Immunoblot analysis of SEPT2 and HSPA5 in iBMDMs after being infected with VSV (MOI = 1), HSV-1 (MOI = 5), or PR8M (MOI = 1) in the absence or presence of 4-PBA (5 mM) for 12 hours. **g.** Immunoblot analysis of SEPT2 in iBMDMs after being transfected with siRNAs targeting PERK, IRE1 and ATF6 and infected with VSV (MOI = 1), HSV-1 (MOI = 5), or PR8M (MOI = 1) for 12 hours. n = 3 in each group (**f, g**). **h.** Validation of *Xbp1*$^{-/-}$ iBMDMs. n = 3 in each group (**h**). **i.** Dual-luciferase reporter assay of sXBP1-binding activity to SEPT2 promoter truncations in HEK-293FT cells. **j.** Dual-luciferase reporter assay of sXBP1-binding activity to WT and mutated 2.5kb SEPT2 promoter in HEK-293FT cells after being cotransfected with different doses of sXBP1 overexpression plasmids. n = 6 in each group (**i, j**). **k.** Chromatin immunoprecipitation assay of sXBP1-binding activity to SEPT2 promoter in iBMDMs after being infected with VSV (MOI = 1) for 12 hours. n = 6 in each group (**k**). **l.** Electrophoretic mobility shift

assay of sXBP1-binding activity to SEPT2 probe. n = 3 in each group (**l**). **m.** qRT-PCR analysis of SEPT2 in PBMCs obtained from healthy individuals (n = 21, 10 males and 11 females), influenza patients without cytokine storm (n = 29, 19 males and 10 females) and influenza patients with cytokine storm (n = 25, 17 males and 8 females). **n, o.** PBMCs obtained from influenza patients with cytokine storm were treated with IXA4 (10 μM) or APY29 (1 μM) for 6 hours. The XBP1 mRNA splicing level (**n**) and the expression of SEPT2 (**o**) were detected. n = 12 in each group (**n, o**). Data are shown as the mean ± s.e.m. (**a, c-e, i-k, n, o**) and the median ± interquartile (**m**). One-way ANOVA followed by Bonferroni post *hoc* test (**a, c-e, i-k**) and Mann-Whitney *U* test (**m-o**) were used for data analysis. Abbreviation: UI, uninfected. EV, empty vector. Source data are provided as a Source Data file.

Supplementary Methods

Plasmid and siRNA transfection

Overexpressing plasmids, siRNAs and their negative controls were constructed or purchased from GenePharma (GenePharma, Shanghai, China). Cell transfection was performed using a LONZA 4D-Nucleofector system according to the manufacturer's instruction. When cell confluency reached 75%, 1×10^6 cells were collected and mixed with 1 μg plasmids or 40 pmol siRNA. The cell-nucleic acid mixture was added into Nucleofector Solution with Supplement and transferred into Nucleocuvette Vessel. The time constant was set to 5 ms. After electroporation, cells were transferred to the plastic dish for further culture. The transfection efficiency was detected after 12 hours (overexpressing plasmid) or 24 hours (siRNA). *In vivo* transfection was performed as previously described[1]. Briefly, nucleic acid (40 μg) were combined with *in vivo*-jetPEI delivery reagent (Polyplus-transfection, NY, USA) in a 5% glucose solution (N/P ratio = 8). The solution was mixed and incubated at room temperature for 30 min, and were then intravenously injected into mice. The transfection efficiency in lung tissue was detected by western blotting. All the primers used for plasmid construction are listed in Supplementary Data 3.

High-content screening

In order to screen the IFN-γ-independent regulatory genes of polarization, IFNGR1-deficient iBMDMs were seeded into 96-well plates, and then siRNA (25 nM) targeting each gene and iNOS promoter::GFP vector were transfected into cells.

Twenty-four hours later, cells were infected with VSV (MOI = 1) or HSV-1 (MOI = 5) for 12 hours. For screening of E3 ubiquitin ligase regulating HSPA5, siRNA (25 nM) targenting each gene and HSPA5::GFP vector were transfected into *Sept2*$^{fl/fl}$ *Lyz2*-Cre PMs, followed by VSV infection (MOI = 1) for 12 hours. After that, cells were fixed with 4% paraformaldehyde (PFA) and stained with DAPI. Images were taken under the 20× and 40× objectives and two detection channels (FITC and DAPI) of the ImageXpress Pico system (Molecular Devices, Shanghai, China). Cells in each well were taken one image under 20× objective and three images under 40× objective for quantitative analysis. The mean GFP fluorescence intensity was calculated based on cell area and GFP fluorescence intensity. Statistical analysis was performed by CellReporterXpress automated imaging analysis software (Molecular Devices). The catalog of two siRNA libraries is listed in Supplementary Data 1 and Supplementary Data 2.

Histopathology analysis

Six mice from each group were randomly selected for histopathology analysis. Lungs were fixed with 10% PFA for more than 24 hours, embedded in paraffin and sectioned at a thickness of 2 μm. Hematoxylin and eosin (H&E) staining was applied to detect inflammation. The one representative slice obtained from each mouse was chose for scoring. Pathology score was determined by assessment of alveolitis, inflammatory cell infiltration and peribronchiolar inflammation. A score of 0 indicated healthy lung; 1= very mild damage; 2 = mild damage; 3 = moderate damage; 4 = severe damage;

and 5 = extremely severe damage. The scoring was performed blinded.

Immunohistochemistry

Lungs were cut into segments, fixed in 10% PFA and embedded in paraffin. Blocks were sectioned at a thickness of 5 μm. After tissue antigen recovery, sections were processed for IHC staining using iNOS antibody (MA5-17139, Invitrogen) at a 1:200 dilution, and then incubated with HRP-tagged secondary antibody (Beyotime, Jiangsu, China). Immunostaining was visualized with 3,3-diaminobenzidine (DAB) substrate. Finally, hematoxylin was used to stain nuclei.

Flow cytometry

PMs obtained from mice were purified using F4/80 antibody (40781, Cell Signaling Technology, Inc., Danvers, MA, USA), and then tagged with fluorescently labeled antibodies as indicated. BD LSR II flow cytometer (BD Biosciences, San Jose, CA, USA) and FlowJo software were used to analyze cell aggregations. The gating strategy of innate immune cell populations in lungs referred to a previous report with minor modification[2]. Mice were perfused with sterile PBS and the left lung lobe was digested into single cell suspensions. Cells were incubated with antibodies against the following markers: PE-Cy7 anti-CD45 (552848), PE anti-Ly6G (561104), FITC anti-CD11b (557396), FITC anti-Ly6C (553104), PE anti-CD11c (561044), APC anti-CD86 (558703), APC anti-CD11b (561690), PE anti-F4/80 (566787), Alexa Fluor 488 anti-CD206 (568806), PerCP-Cy5.5 anti-Ly6C (560525), BV786 anti-CD103

(744679), Anti-NK1.1 (560515), APC-Cy7 anti-siglecF (565527), FITC anti-B220 (553088), APC anti-CD3e (553066), PE-Cy7 anti-CD8a (552877), PerCP-Cy5.5 anti-CD8a (551162) and FITC anti-CD4 (553729) (BD Biosciences). All antibodies were used at a dilution of 1:200. iNOS (MA5-17139), CD80 (12-0801-82), CD86 (11-0862-82) (Invitrogen) and Arg-1 (PA5-85267), CD163 (61-1631-82), CD206 (53-2061-82) (Invitrogen) were used as M1-like and M2-like macrophage markers, respectively.

qRT-PCR

Total RNA were isolated using Trizol reagent (Invitrogen). Purified RNA (1 μg) was reverse-transcribed to cDNA in 20 μL reaction using a SYBR PrimeScript RT-PCR Kit (Takara, Otsu, Shiga, Japan). qRT-PCR was performed using a TB Green Premix ExTaq II Kit (Takara) on Bio-Rad CFX-96 system (Bio-Rad, Hercules, CA, USA). cDNA (2 μL) was amplified in 25 μL reaction containing 12.5 μL TB Green Premix ExTaq II and 0.4 μM forward/reverse primers. The cycling conditions were as follows: predenaturation (95 ℃, 30 s), denaturation (95 ℃, 5 s, 39 cycles) and extension (60 ℃, 30 s). Results were normalized to GAPDH mRNA levels according to the $2^{-\Delta\Delta Ct}$ method. Primer sequences were obtained from PrimerBank-Harvard University (https://pga.mgh.harvard.edu/primerbank/). XBP1 splicing was detected by qRT-PCR as previously described[3]. Primers for quantification of spliced XBP1 (s) and total XBP1 (t, common region of spliced/unspliced XBP1) are listed in Supplementary Data 3.

Western blotting and coimmunoprecipitation

Cells were harvested and lysed with lysis buffer [20 mM Tris (pH 7.5), 150 mM NaCl, 1% Triton X-100, sodium pyrophosphate, β-glycerophosphate, EDTA, Na_3VO_4 and leupeptin] (P0013, Beyotime) containing protease inhibitor cocktail. Cell lysates were fractionated by sodium dodecyl sulfate polyacrylamide gel electrophoresis (SDS-PAGE) and transferred to polyvinylidene fluoride (PVDF) membranes (Millipore, Bedford, MA, USA). Blots were probed with the indicated antibodies: 1/400 anti-HSPA5 (NBP1-54318), 1/400 anti-ATF6α (NBP2-76329), 1/400 anti-CHOP (NB600-1335), 1/400 anti-ATF4 (NB100-852), 1/400 anti-SCNN1B (NBP2-59383), 1/400 anti-ATAT1 (NBP2-48860), 1/1,000 anti-GAPDH (NBP2-27103) (Novus Biologicals, Englewood, CO, USA), 1/400 anti-SEPT2 (sc-514206), 1/500 anti-Ubiquitin (sc-8017), 1/400 anti-SEPT6 (sc-514781) (Santa Cruz Biotechnology, Santa Cruz, CA, USA), 1/400 anti-Phospho-PERK (3179), 1/400 anti-PERK (3192), 1/400 anti-sXBP1 (40435), 1/400 anti-acetyl-NF-κB p65 (Lys310) (3045), 1/400 anti-Phospho-eIF2α (9721), 1/400 anti-eIF2α (9722), 1/400 anti-IRE1α (3294), 1/400 anti-RIG-I (3743), 1/400 anti-cGAS (31659), 1/400 anti-MDA5 (5321), 1/400 anti-TRAF2 (4712), 1/400 anti-Phospho-c-Jun (2994), 1/400 anti-c-Jun (9165), 1/400 anti-Phospho-IκBα (2859), 1/400 anti-IκBα (4814), 1/400 anti-GRK2 (74761) (Cell Signaling Technology), 1/400 anti-TLR3 (PA5-20183), 1/400 anti-TLR9 (PA5-20203), 1/400 anti-MAVS (PA5-20348), 1/400 anti-IFNAR1 (MA5-42390), 1/400 anti-IFNAR2 (PA5-76100), 1/400 anti-Phospho-IRE1α (PA5-105424), 1/400 anti-

SEPT7 (PA5-56181), 1/400 anti-SEPT9 (PA5-100077), 1/400 anti-GSDMD (MA5-44666), 1/400 anti-JNK1/2 (AHO1362), 1/400 anti-Phospho-JNK1/2 (700031), 1/400 anti-WDTC1 (PA5-113155) (Thermo Fisher Scientific), 1/400 anti-V5 (AF2894), 1/400 anti-HA (AF2858), 1/400 anti-Myc (AF2864), 1/400 anti-Flag (AF519), 1/200 pan Acetyl-Lysine antibody (AF5632), 1/200 pan Di-Methyl-Lysine antibody (AF5701), 1/200 pan Phospho-Serine/Threonine antibody (AF5725) (Beyotime). Antibodies were diluted in in 1% BSA (pan Phospho-Serine/Threonine antibody) or 5% skim milk (others). For coimmunoprecipitation (except ubiquitin immunoprecipitation), cell lysates were obtained according to the standard cell lysis method. Lysates were centrifuged and the supernatants were incubated with appropriate antibodies and Protein A-Agarose (Santa Cruz Biotechnology) overnight at 4 ℃. Precipitated protein complex was mixed with 5 × SDS Loading Buffer and boiled at 98 ℃ for 3 min, followed by immunoblotting with indicated antibodies. The uncropped blots are provided in the Source Data file.

Tandem ubiquitin binding entity analysis

Tandem ubiquitin binding entities (TUBEs, UM401M, LifeSensors, Malvern, PA, USA) were used for ubiquitin immunoprecipitation. Cells were harvested and lysed in specific cell lysis buffer [50 mM Tris-HCl (pH 7.5), 0.15 M NaCl, 1 mM EDTA, 1% NP-40 and 10% glycerol]. Then, equilibrated magnetic-TUBEs were added into cell lysate (100 μL resin/1 mg protein) and incubated for 2 hours at 4 ℃ on a rocker platform. After washing for three times with TBST, beads were collected using a

magnetic stand for western blotting analysis.

Enzyme linked immunosorbent assay

Cell culture supernatants were purified by centrifugation, diluted with Assay Diluent A (contains 0.09% sodium azide) and then assayed by enzyme linked immunosorbent assay (ELISA). TNF-α (A43658), IL-1β (A42894), IL-6 (A43656), IL-12 p40/70 (EMIL12B), IFN-γ (BMS606-2), IFN-α (BMS6027) and IFN-β (A47435) ELISA kits were purchased from e-Biosciences (Thermo Fisher Scientific). Bronchoalveolar lavage fluid samples obtained from virus-infected mice were diluted 10-fold with Assay Diluent A. The concentration of each cytokine was calculated against a standard curve. All diluted samples fell within the standard curve.

RNA-sequencing

RNA-seq was performed by Shanghai Majorbio Bio-pharm Technology Co.,Ltd. Total RNA extracted from *Sept2*$^{fl/fl}$ and *Sept2*$^{fl/fl}$ *Lyz2*-Cre PMs infected with VSV or HSV-1 was used to construct the sequencing library. RNA-seq transcriptome library was prepared following TruSeq RNA sample preparation Kit from Illumina (San Diego, CA) using 1 μg of total RNA. Double-stranded cDNA was synthesized using a SuperScript double-stranded cDNA synthesis kit (Invitrogen) with random hexamer primers (Illumina). Then the synthesized cDNA was subjected to end-repair, phosphorylation and ‘A’ base addition according to Illumina’s library construction protocol. Libraries were size selected for cDNA target fragments of 300 bp on 2%

Low Range Ultra Agarose followed by PCR amplification using Phusion DNA polymerase (New England Biolabs) for 15 PCR cycles. After quantified by TBS380, paired-end RNA-seq library was sequenced with the Illumina HiSeq xten/NovaSeq 6000 sequencer (2 ×150 bp read length). The raw paired-end reads were trimmed and quality controlled by SeqPrep and Sickle with default parameters. Then clean reads were separately aligned to Mus_musculus GRCm39 reference genome with orientation mode using HISAT2 software. The mapped reads of each sample were assembled by StringTie. Differential expression analysis and functional enrichment were performed using the DESeq2 software[4] on the Majorbio Cloud Platform. The filtering threshold is $|\log_2 FC| >= 1$ & $Padjust < 0.01$. The RNA-seq data are available in Gene Expression Omnibus at GSE213863.

Transmission electron microscopy

PMs obtained from *Sept2*$^{fl/fl}$ and *Sept2*$^{fl/fl}$ *Lyz2*-Cre mice were infected with VSV (MOI = 1) or HSV-1 (MOI = 5) for 12 hours. Cells were collected and fixed with 2% glutaraldehyde, and then postfixed with 1% OsO_4 for 2 hours. After washing with distilled water, the samples were gradually dehydrated with ethanol, transferred to propylene oxide and embedded in epon. Ultra-thin slicer was used to cut the samples into 70 nm slices. The slices were dyed with 3% uranium acetate-lead citrate. Imaging was performed with a JEOL JEM-1400 plus transmission electron microscopy (magnifications ranging from × 4.0k to × 12.0k, voltage: 80.0 kV). At least 10 images of each sample were acquired for subcellular structure.

Detection of the accumulation of unfolded proteins

NTPAN-MI probe was resynthesized as previously described[5] and used to analyze the accumulation of unfolded proteins. The resynthesis process was performed strictly according to the reference. The pure product was obtained after silica-gel chromatography in 62% yield as a yellow solid and identified by ^{1}H NMR and ^{13}C NMR. For cell staining, NTPAN-MI was dissolved in DMSO as 2 mM stocks and diluted to 50 μM before use. Cells were treated with 50 μM NTPAN-MI for 30 min at room temperature and subsequently washed. DAPI was used to stain nuclei. Before imaging, cells were fixed with 4% PFA for 20 min. Images were acquired on a Leica DMi8 inverted fluorescence microscope. The fluorescence of NTPAN-MI was detected in the 527 nm range with excitation at 405 nm. DAPI fluorescence was monitored in the 460 nm range with excitation at 350 nm.The mean fluorescence intensity was quantitated by image J software.

ROS production detection

H_2DCFDA (D399, Invitrogen) was used for oxidative stress measurement according to the manufacturer's instruction. Briefly, PMs obtained from *Sept2*$^{fl/fl}$ and *Sept2*$^{fl/fl}$ *Lyz2*-Cre mice were infected with VSV (MOI = 1) for the indicated times. The 1400W·dihydrochloride (100 μM) was used to inhibit iNOS activity. H_2DCFDA (10 μM) was added into the cell culture medium and incubated at 37 ℃ for 20 min. The reactive oxygen species production was determined by detecting the fluorescence in

the 517-527 nm range with excitation in the 492-495 nm range.

Identification of acetylated and ubiquitinated lysine residues by HPLC-MS/MS

PMs obtained from *Sept2*$^{fl/fl}$ and *Sept2*$^{fl/fl}$ *Lyz2*-Cre mice were infected with VSV (MOI = 1) for 12 hours. To obtain the protein samples, cells were lysed with lysis buffer (8 M urea containing protease inhibitor cocktail) and centrifugated at 12,000× g at 4 ℃ for 10 min. The supernatant was treated with trypsin at a ratio of 1:50 overnight for digestion. Anti-lysine acetylation antibody beads and anti-lysine ubiquitin antibody beads were used to enrich acetylated and ubiquitinated peptides, respectively. Next, enriched peptides were separated by the EASY-nLC HPLC system and detected by tandem mass spectrometry in the Q-Exactive mass spectrometer (Thermo Fisher Scientific). Data were analyzed using Mascot software. The increase of 42.01 Da and 114.1 Da of lysine residue was calculated to determine acetylation and ubiquitination, respectively.

Duolink PLA assay

Duolink PLA assay was performed according to the kit manual (DUO96010 and DUO96020, Sigma-Aldrich, St. Louis, MO, USA). In brief, VSV-infected cells were fixed with 4% PFA on slides for 20 min and permeabilized with 0.2% Triton X-100. Next, add 40 μL of blocking solution to the slide and incubate the slide at 37 ℃ for 60 min. Dilute the oligo-conjugated primary antibodies (Red Oligo A and Red Oligo B for anti-HSPA5 antibody and anti-Ubiquitin antibody, Green Oligo C and Green Oligo

D for anti-HSPA5 antibody and pan Acetyl-Lysine antibody) in the Probemaker PLA Probe Diluent and add the primary antibody solutions to each slide. The slides were incubated at 4 ℃ for 8 hours, followed by ligation, amplification and detection. After final washing, the acetylation and ubiquitination levels of HSPA5 were identified by fluorescence intensity.

Protein complex structure analysis

The structures of ATAT1 and SCNN1B were first predicted by AlphaFold2, and the inactive sites were removed. Since HSPA5 has template information with high similarity, the prediction model of HSPA5 was obtained by homology modeling with SwissDock. Next, ZDOCK and HADDOCK were used to achieve protein-protein rigid docking, and RosettaDock was used to achieve protein-protein flexible docking. The preliminary conformation of the global docking was optimized with Rosetta in two rounds. The results were quality checked according to the Rosetta evaluation metric. After screening by the scoring function, biochemical experimental data and empirical knowledge, the final models of ATAT1/HSPA5 and SCNN1B/HSPA5 complexes were obtained. The binding interfaces of the two complexes were comprehensively characterized and systematically analyzed using interaction analysis platforms PDBsum and PLIP.

Isothermal titration calorimetry

ITC was used to identify the affinity of ATAT1 or SCNN1B to HSPA5 as previously

described[6]. Purified proteins were transferred to buffer containing 20 mM HEPES (pH 7.5), 100 mM NaCl, and 2 mM β-mercaptoethanol by HiTrap desalting column (GE healthcare, Atlanta, GA, USA). Titrations were performed by using a Malvern Microcal PEAQ-ITC calorimeter at room temperature. Data were analyzed using the PEAQ-ITC analysis software.

Surface plasmon resonance

SPR was used to identify the affinity of ATAT1 or SCNN1B to HSPA5 as previously described[7]. Purified HSPA5 protein (20 μg/mL, in 10 mM sodium acetate buffer, pH 5.0) was coupled on a CM5 Chip (GE Healthcare) using amine coupling method. Different doses of ATAT1 or SCNN1B proteins (0 to 2,000 nM) were injected for association and dissociation analysis. The dissociation rate constants were calculated using steady state affinity obtained for each enzyme concentration.

Fluorescent resonance energy transfer

FRET system was built and performed as shown in Figure S6J-K following a previous report with partial modification[8]. Briefly, CMV-HSPA5(TAG)-ATAT1-YFP (or CMV-HSPA5(TAG)-SCNN1B-YFP) was transfected into *Atat1*$^{-/-}$ *Scnn1b*$^{-/-}$ iBMDMs, followed by transfection of different doses of SCNN1B (or ATAT1). A fluorescent amino acid ANAP was incorporated into Phenylalanine 452 of HSPA5. The fluorescence of ANAP-HSPA5 was detected in the 410-530 nm range with excitation at 405 nm, and YFP-ATAT1 (or YFP-SCNN1B) fluorescence was monitored in the

550-620 nm range with excitation at both 405 and 488 nm. FRET signals arising from binding of HSPA5 to ATAT1 (or SCNN1B) were monitored in the 550-620 nm range with excitation of ANAP at 405 nm. FRET ratio (I_{YFP}/I_{ANAP}) was recorded from single live cell image at the indicated times.

Dual-luciferase reporter assay

HEK-293FT cells were seeded on 24-well plate and cultured overnight to reach 75% confluency. Luciferase reporter plasmids (300 ng) and internal control plasmid pRL-SV40 (10 ng) were cotransfected into cells for 24 hours. Afterwards, cells were lysed for dual-luciferase detection. The relative luciferase activity was measured by firefly luciferase luminescence divided by renilla luciferase luminescence. All the primers used for luciferase reporter plasmids construction are listed in Supplementary Data 3.

Chromatin immunoprecipitation assay

ChIP assay was performed using SimpleChIP Plus Enzymatic Chromatin IP Kit (9004S, Cell Signaling Technology). Cells were fixed with 1% formaldehyde to cross-link histone and non-histone proteins to DNA, and then pellet nuclei was collected by centrifugation at 2,000 × g for 5 min at 4 ℃. Next, micrococcal nuclease (0.5 μL per 4 × 10^6 cells) was added to the pellet nuclei. The lysate was incubated at 37 ℃ for 20 min with frequent mixing to digest chromatin to a length of approximately 150-900 bp. Then lysate was subjected to sonication to break nuclear membrane. Incubate samples for 30 s on wet ice between pulses. Afterwards, clarify lysate by

centrifugation at 9,400×g for 10 min at 4 ℃. Finally, digested chromatin was incubated with magnetic beads and anti-sXBP1 antibody (40435, Cell Signaling Technology) in rotation overnight at 4 ℃. Immunoprecipitated chromatin DNA was eluted and quantified by qRT-PCR.

Electrophoretic mobility shift assay

Electrophoretic mobility shift assay was performed using fluorescence-based electrophoretic mobility shift assay kit (Molecular Probe, Thermo Fisher Scientific). Purified sXBP1 protein and SEPT2 DNA probes were synthesized by Sangon (Shanghai, China). sXBP1-SEPT2 complex was identified by electrophoresis on a 6% polyacrylamide gel. The sequences of SEPT2 DNA probes are listed in Supplementary Data 3.

Supplementary References

1. Fu, B. *et al.* MiR-342 controls Mycobacterium tuberculosis susceptibility by modulating inflammation and cell death. *EMBO Rep* **22**, e52252 (2021).

2. Nair, S. *et al.* Irg1 expression in myeloid cells prevents immunopathology during M. tuberculosis infection. *J Exp Med* **215**, 1035-1045 (2018).

3. Yoon, S.B. *et al.* Real-time PCR quantification of spliced X-box binding protein 1 (XBP1) using a universal primer method. *PLoS One* **14**, e0219978 (2019).

4. Love, M.I., Huber, W. & Anders, S. Moderated estimation of fold change and dispersion for RNA-seq data with DESeq2. *Genome biology* **15**, 550 (2014).

5. Owyong, T.C. *et al.* A Molecular Chameleon for Mapping Subcellular Polarity in an Unfolded Proteome Environment. *Angewandte Chemie* **59**, 10129-10135 (2020).

6. Lin, Y. *et al.* Phytophthora sojae effector Avr1d functions as an E2 competitor and inhibits ubiquitination activity of GmPUB13 to facilitate infection. *Proc Natl Acad Sci U S A* **118**, e2018312118 (2021).

7. Ghilarducci, K. *et al.* Functional interaction of ubiquitin ligase RNF167 with UBE2D1 and UBE2N promotes ubiquitination of AMPA receptor. *The FEBS journal* **288**, 4849-4868 (2021).

8. Park, S.H., Ko, W., Lee, H.S. & Shin, I. Analysis of Protein-Protein Interaction in a Single Live Cell by Using a FRET System Based on Genetic Code Expansion Technology. *Journal of the American Chemical Society* **141**, 4273-4281 (2019).
