# Supplementary material for: SEPTIN2 suppresses an IFN-γ-independent, proinflammatory macrophage activation pathway
Source: Nat Commun. 2023 Nov 17;14:7441. doi: 10.1038/s41467-023-43283-2 (PMC10656488; doi:10.1038/s41467-023-43283-2)
Supplement: Supplementary file 6 — Reporting Summary [file 41467_2023_43283_MOESM6_ESM.pdf]

nature portfolio

Corresponding author(s): Haibo Wu, Xiaoyuan Lin, Yang Luo

Last updated by author(s): Oct 24, 2023

Reporting Summary

Nature Portfolio wishes to improve the reproducibility of the work that we publish. This form provides structure for consistency and transparency in reporting. For further information on Nature Portfolio policies, see our Editorial Policies and the Editorial Policy Checklist.

Statistics

For all statistical analyses, confirm that the following items are present in the figure legend, table legend, main text, or Methods section.

n/a	Confirmed	
☐	☒	The exact sample size (*n*) for each experimental group/condition, given as a discrete number and unit of measurement
☐	☒	A statement on whether measurements were taken from distinct samples or whether the same sample was measured repeatedly
☐	☒	The statistical test(s) used AND whether they are one- or two-sided<br>*Only common tests should be described solely by name; describe more complex techniques in the Methods section.*
☐	☒	A description of all covariates tested
☐	☒	A description of any assumptions or corrections, such as tests of normality and adjustment for multiple comparisons
☐	☒	A full description of the statistical parameters including central tendency (e.g. means) or other basic estimates (e.g. regression coefficient) AND variation (e.g. standard deviation) or associated estimates of uncertainty (e.g. confidence intervals)
☐	☒	For null hypothesis testing, the test statistic (e.g. *F*, *t*, *r*) with confidence intervals, effect sizes, degrees of freedom and *P* value noted<br>*Give P values as exact values whenever suitable.*
☒	☐	For Bayesian analysis, information on the choice of priors and Markov chain Monte Carlo settings
☒	☐	For hierarchical and complex designs, identification of the appropriate level for tests and full reporting of outcomes
☐	☒	Estimates of effect sizes (e.g. Cohen's *d*, Pearson's *r*), indicating how they were calculated

Our web collection on statistics for biologists contains articles on many of the points above.

Software and code

Policy information about availability of computer code

Data collection	No software was used.
Data analysis	GraphPad Prism 8.3.0 for statistics; ImageJ 1.52a for image analysis; FlowJo 10.6.2 for cell aggregation analysis; GPP sgRNA Designer for designing CRISPR sgRNAs; Primer Premier 5.00 for designing primers used for plasmid construction; Majorbio Cloud Platform for RNA-seq analysis; PDBsum and PLIP for protein complex structure analysis; PEAQ-ITC software for ITC analysis;CellReporterXpress automated imaging analysis software for HCS analysis

For manuscripts utilizing custom algorithms or software that are central to the research but not yet described in published literature, software must be made available to editors and reviewers. We strongly encourage code deposition in a community repository (e.g. GitHub). See the Nature Portfolio guidelines for submitting code & software for further information.

Data

Policy information about availability of data

All manuscripts must include a data availability statement. This statement should provide the following information, where applicable:

- Accession codes, unique identifiers, or web links for publicly available datasets
- A description of any restrictions on data availability
- For clinical datasets or third party data, please ensure that the statement adheres to our policy

The RNA-seq data generated in this study is publicly available in Gene Expression Omnibus at GSE213863 [https://www.ncbi.nlm.nih.gov/geo/query/acc.cgi?acc=GSE213863]. Source data are provided with this paper.

Research involving human participants, their data, or biological material

Policy information about studies with human participants or human data. See also policy information about sex, gender (identity/presentation), and sexual orientation and race, ethnicity and racism.

Reporting on sex and gender	healthy individuals (n = 21, 10 males and 11 females), influenza patients without cytokine storm (n = 29, 19 males and 10 females) and patients with cytokine storm (n = 25, 17 males and 8 females)
Reporting on race, ethnicity, or other socially relevant groupings	We did not collect race, ethnicity or other social variables.
Population characteristics	healthy individuals (n = 21, aged 32.13 ± 8.57 years), influenza patients without cytokine storm (n = 29, aged 34.15 ± 6.34 years) and patients with cytokine storm (n = 25, aged 37.59 ± 4.12 years)
Recruitment	All blood samples were obtained from Chongqing Public Health Medical Center between December 2018 and February 2022. Both influenza patients with/without cytokine storm were confirmed as being infected with Influenza A virus by qRT-PCR. Samples were divided into 3 groups according to the source of healthy people, influenza patients without cytokine storm and influenza patients with cytokine storm.
Ethics oversight	The ethics committee of Chongqing Public Health Medical Center approved this consent procedure. This study was compliant with the “Guidance of the Ministry of Science and Technology (MOST) for the Review and Approval of Human Genetic Resources”.

Note that full information on the approval of the study protocol must also be provided in the manuscript.

Field-specific reporting

Please select the one below that is the best fit for your research. If you are not sure, read the appropriate sections before making your selection.

☒ Life sciences ☐ Behavioural & social sciences ☐ Ecological, evolutionary & environmental sciences

For a reference copy of the document with all sections, see nature.com/documents/nr-reporting-summary-flat.pdf

Life sciences study design

All studies must disclose on these points even when the disclosure is negative.

Sample size	Sample size was based on empirical data from pilot experiments. For flow cytometry, immunoblot and immunofluorescence, n = 3. For other data obtained from cell and animal experiments, n = 6 or n = 9. The sample size was indicated in the figure legends. For experiments using human samples, sample size was limited by the patient samples that we could obtain and was indicated in the figure legends and the Methods section.
Data exclusions	No data were excluded from the analysis.
Replication	Replication details are indicated in "Sample size" and the figure legends.
Randomization	Mice of similar ages were randomly allocated into different groups. All mice were age- and sex-matched in each experiment. Human blood samples were divided into 3 groups according to the source of healthy people, influenza patients without cytokine storm and influenza patients with cytokine storm.
Blinding	The investigators were blinded during data collection and analysis.

Reporting for specific materials, systems and methods

We require information from authors about some types of materials, experimental systems and methods used in many studies. Here, indicate whether each material, system or method listed is relevant to your study. If you are not sure if a list item applies to your research, read the appropriate section before selecting a response.

Materials & experimental systems

n/a	Involved in the study	
☐	☒	Antibodies
☐	☒	Eukaryotic cell lines
☒	☐	Palaeontology and archaeology
☐	☒	Animals and other organisms
☒	☐	Clinical data
☒	☐	Dual use research of concern
☒	☐	Plants

Methods

n/a	Involved in the study	
☒	☐	ChIP-seq
☐	☒	Flow cytometry
☒	☐	MRI-based neuroimaging

Antibodies

Antibodies used

For flow cytometry, PE-Cy7 anti-CD45 (552848), PE anti-Ly6G (561104), FITC anti-CD11b (557396), FITC anti-Ly6C (553104), PE anti-CD11c (561044), APC anti-CD86 (558703), APC anti-CD11b (561690), PE anti-F4/80 (566787), Alexa Fluor 488 anti-CD206 (568806), PerCP-Cy5.5 anti-Ly6C (560525), BV786 anti-CD103 (744679), Anti-NK1.1 (560515), APC-Cy7 anti-siglecF (565527), FITC anti-B220 (553088), APC anti-CD3e (553066), PE-Cy7 anti-CD8a (552877), PerCP-Cy5.5 anti-CD8a (551162) and FITC anti-CD4 (553729) (BD Biosciences), iNOS (MA5-17139), CD80 (12-0801-82), CD86 (11-0862-82), Arg-1 (PA5-85267), CD163 (61-1631-82) and CD206 (53-2061-82) (Invitrogen). For western blotting, coimmunoprecipitation and chromatin immunoprecipitation assay, anti-HSPA5 (NBP1-54318), anti-ATF6α (NBP2-76329), anti-CHOP (NB600-1335), anti-ATF4 (NB100-852), anti-SCNN1B (NBP2-59383), anti-ATAT1 (NBP2-48860), anti-GAPDH (NBP2-27103) (Novus Biologicals), anti-SEPT2 (sc-514206), anti-Ubiquitin (sc-8017), anti-SEPT6 (sc-514781) (Santa Cruz Biotechnology), anti-Phospho-PERK (3179), anti-PERK (3192), anti-sXBP1 (40435), anti-acetyl-NF-κB p65 (Lys310) (3045), anti-Phospho-eIF2α (9721), anti-eIF2α (9722), anti-IRE1α (3294), anti-RIG-I (3743), anti-cGAS (31659), anti-MDA5 (5321), anti-TRAF2 (4712), anti-Phospho-c-Jun (2994), anti-c-Jun (9165), anti-Phospho-IκBα (2859), anti-IκBα (4814), anti-GRK2 (74761) (Cell Signaling Technology), anti-TLR3 (PA5-20183), anti-TLR9 (PA5-20203), anti-MAVS (PA5-20348), anti-IFNAR1 (MA5-42390), anti-IFNAR2 (PA5-76100), anti-Phospho-IRE1α (PA5-105424), anti-SEPT7 (PA5-56181), anti-SEPT9 (PA5-100077), anti-GSDMD (MA5-44666), anti-JNK1/2 (AHO1362), anti-Phospho-JNK1/2 (700031), anti-WDTC1 (PA5-113155) (Thermo Fisher Scientific), anti-V5 (AF2894), anti-HA (AF2858), anti-Myc (AF2864), anti-Flag (AF519), pan Acetyl-Lysine antibody (AF5632), pan Di-Methyl-Lysine antibody (AF5701), pan Phospho-Serine/Threonine antibody (AF5725) (Beyotime)

Validation

All antibodies used in the study are commercial antibodies. The manufacturer's websites with validation statements are as follows: PE-Cy7 anti-CD45 (552848, https://www.bdbiosciences.com/zh-cn/products/reagents/flow-cytometry-reagents/research-reagents/single-color-antibodies-ruo/pe-cy-7-rat-anti-mouse-cd45.552848), PE anti-Ly6G (561104, https://www.bdbiosciences.com/zh-cn/products/reagents/flow-cytometry-reagents/research-reagents/single-color-antibodies-ruo/pe-rat-anti-mouse-ly-6g.561104), FITC anti-CD11b (557396, https://www.bdbiosciences.com/zh-cn/products/reagents/flow-cytometry-reagents/research-reagents/single-color-antibodies-ruo/fitc-rat-anti-cd11b.557396), FITC anti-Ly6C (553104, https://www.bdbiosciences.com/zh-cn/products/reagents/flow-cytometry-reagents/research-reagents/single-color-antibodies-ruo/fitc-rat-anti-mouse-ly-6c.553104), PE anti-CD11c (561044, https://www.bdbiosciences.com/zh-cn/products/reagents/flow-cytometry-reagents/research-reagents/single-color-antibodies-ruo/pe-hamster-anti-mouse-cd11c.561044), APC anti-CD86 (558703, https://www.bdbiosciences.com/zh-cn/products/reagents/flow-cytometry-reagents/research-reagents/single-color-antibodies-ruo/apc-rat-anti-mouse-cd86.558703), APC anti-CD11b (561690, https://www.bdbiosciences.com/zh-cn/products/reagents/flow-cytometry-reagents/research-reagents/single-color-antibodies-ruo/apc-rat-anti-cd11b.561690), PE anti-F4/80 (566787, https://www.bdbiosciences.com/zh-cn/products/reagents/flow-cytometry-reagents/research-reagents/single-color-antibodies-ruo/apc-rat-anti-mouse-f4-80.566787), Alexa Fluor 488 anti-CD206 (568806, https://www.bdbiosciences.com/zh-cn/products/reagents/flow-cytometry-reagents/research-reagents/single-color-antibodies-ruo/alexa-fluor-488-rat-anti-mouse-cd206.568806), PerCP-Cy5.5 anti-Ly6C (560525, https://www.bdbiosciences.com/zh-cn/products/reagents/flow-cytometry-reagents/research-reagents/single-color-antibodies-ruo/percp-cy-5-5-rat-anti-mouse-ly-6c.560525), BV786 anti-CD103 (744679, https://www.bdbiosciences.com/zh-cn/products/reagents/flow-cytometry-reagents/research-reagents/single-color-antibodies-ruo/bv786-mouse-anti-rat-integrin-e2-cd103.744679), Anti-NK1.1 (560515, https://www.bdbiosciences.com/zh-cn/products/reagents/flow-cytometry-reagents/research-reagents/single-color-antibodies-ruo/alexa-fluor-700-mouse-anti-mouse-nk1-1.560515), APC-Cy7 anti-siglecF (565527, https://www.bdbiosciences.com/zh-cn/products/reagents/flow-cytometry-reagents/research-reagents/single-color-antibodies-ruo/apc-cy-7-rat-anti-mouse-siglec-f.565527), FITC anti-B220 (553088, https://www.bdbiosciences.com/zh-cn/products/reagents/flow-cytometry-reagents/research-reagents/single-color-antibodies-ruo/fitc-rat-anti-mouse-cd45r-b220.553088), APC anti-CD3e (553066, https://www.bdbiosciences.com/zh-cn/products/reagents/flow-cytometry-reagents/research-reagents/single-color-antibodies-ruo/apc-hamster-anti-mouse-cd3e.553066), PE-Cy7 anti-CD8a (552877, https://www.bdbiosciences.com/zh-cn/products/reagents/flow-cytometry-reagents/research-reagents/single-color-antibodies-ruo/pe-cy-7-rat-anti-mouse-cd8a.552877), PerCP-Cy5.5 anti-CD8a (551162, https://www.bdbiosciences.com/zh-cn/products/reagents/flow-cytometry-reagents/research-reagents/single-color-antibodies-ruo/percp-cy-5-5-rat-anti-mouse-cd8a.551162) and FITC anti-CD4 (553729, https://www.bdbiosciences.com/zh-cn/products/reagents/flow-cytometry-reagents/research-reagents/single-color-antibodies-ruo/fitc-rat-anti-mouse-cd4.553729), iNOS (MA5-17139, https://www.thermofisher.cn/cn/zh/antibody/product/iNOS-Antibody-clone-4E5-Monoclonal/MA5-17139), CD80 (12-0801-82, https://www.thermofisher.cn/cn/zh/antibody/product/CD80-B7-1-Antibody-clone-16-10A1-Monoclonal/12-0801-82), CD86 (11-0862-82, https://www.thermofisher.cn/cn/zh/antibody/product/CD86-B7-2-Antibody-clone-GL1-Monoclonal/11-0862-82) and Arg-1 (PA5-85267, https://www.thermofisher.cn/cn/zh/antibody/product/Arginase-1-Antibody-Polyclonal/PA5-85267), CD163 (61-1631-82, https://www.thermofisher.cn/cn/zh/antibody/product/CD163-Antibody-clone-TNKUPJ-Monoclonal/61-1631-82), CD206 (53-2061-82, https://www.thermofisher.cn/cn/zh/antibody/product/CD206-MMR-Antibody-clone-MR6F3-Monoclonal/53-2061-82), anti-HSPA5 (NBP1-54318, https://www.novusbio.com/products/grp78-hspa5-antibody_nbp1-54318), anti-ATF6α (NBP2-76329, https://www.novusbio.com/products/atf6-antibody-2358c_nbp2-76329), anti-CHOP (NB600-1335, https://www.novusbio.com/products/gadd153-chop-antibody-9c8_nb600-1335), anti-ATF4 (NB100-852, https://www.novusbio.com/products/atf4-antibody_nb100-852),

anti-SCNN1B (NBP2-59383, https://www.novusbio.com/products/epithelial-sodium-channel-beta-antibody-14e10_nbp2-59383), anti-ATAT1 (NBP2-48860, https://www.novusbio.com/products/atat1-antibody_nbp2-48860), anti-GAPDH (NBP2-27103, https://www.novusbio.com/products/gapdh-antibody-13h12_nbp2-27103), anti-SEPT2 (sc-514206, https://www.scbt.com/zh/p/septin-2-antibody-f-1), anti-Ubiquitin (sc-8017, https://www.scbt.com/zh/p/ubiquitin-antibody-p4d1), anti-SEPT6 (sc-514781, https://www.scbt.com/zh/p/septin-6-antibody-b-8), anti-Phospho-PERK (3179, https://www.cellsignal.cn/products/primary-antibodies/phospho-perk-thr980-16f8-rabbit-mab/3179), anti-PERK (3192, https://www.cellsignal.cn/products/primary-antibodies/perk-c33e10-rabbit-mab/3192), anti-sXBP1 (40435, https://www.cellsignal.cn/products/primary-antibodies/xbp-1s-e9v3e-rabbit-mab/40435), anti-acetyl-NF-κB p65 (Lys310) (3045, https://www.cellsignal.cn/products/primary-antibodies/acetyl-nf-kb-p65-lys310-antibody/3045), anti-Phospho-eIF2α (9721, https://www.cellsignal.cn/products/primary-antibodies/phospho-eif2a-ser51-antibody/9721), anti-eIF2α (9722, https://www.cellsignal.cn/products/primary-antibodies/eif2a-antibody/9722), anti-IRE1α (3294, https://www.cellsignal.cn/products/primary-antibodies/ire1a-14c10-rabbit-mab/3294), anti-RIG-I (3743, https://www.cellsignal.cn/products/primary-antibodies/rig-i-d14g6-rabbit-mab/3743), anti-cGAS (31659, https://www.cellsignal.cn/products/primary-antibodies/cgas-d3o8o-rabbit-mab/31659), anti-MDA5 (5321, https://www.cellsignal.cn/products/primary-antibodies/mda-5-d74e4-rabbit-mab/5321), anti-TRAF2 (4712, https://www.cellsignal.cn/products/primary-antibodies/traf2-antibody/4712), anti-Phospho-c-Jun (2994, https://www.cellsignal.cn/products/primary-antibodies/phospho-c-jun-ser243-antibody/2994), anti-c-Jun (9165, https://www.cellsignal.cn/products/primary-antibodies/c-jun-60a8-rabbit-mab/9165), anti-Phospho-IκBα (2859, https://www.cellsignal.cn/products/primary-antibodies/phospho-ikba-ser32-14d4-rabbit-mab/2859), anti-IκBα (4814, https://www.cellsignal.cn/products/primary-antibodies/ikba-l35a5-mouse-mab-amino-terminal-antigen/4814), anti-GRK2 (74761, https://www.cellsignal.cn/products/primary-antibodies/grk2-antibody/74761), anti-TLR3 (PA5-20183, https://www.thermofisher.cn/cn/zh/antibody/product/TLR3-Antibody-Polyclonal/PA5-20183), anti-TLR9 (PA5-20203, https://www.thermofisher.cn/cn/zh/antibody/product/TLR9-Antibody-Polyclonal/PA5-20203), anti-MAVS (PA5-20348, https://www.thermofisher.cn/cn/zh/antibody/product/MAVS-Antibody-Polyclonal/PA5-20348), anti-IFNAR1 (MA5-42390, https://www.thermofisher.cn/cn/zh/antibody/product/IFNAR1-Antibody-clone-A5-A3-Monoclonal/MA5-42390), anti-IFNAR2 (PA5-76100, https://www.thermofisher.cn/cn/zh/antibody/product/IFNAR2-Antibody-Polyclonal/PA5-76100), anti-Phospho-IRE1α (PA5-105424, https://www.thermofisher.cn/cn/zh/antibody/product/Phospho-IRE1-alpha-Ser724-Antibody-Polyclonal/PA5-105424), anti-SEPT7 (PA5-56181, https://www.thermofisher.cn/cn/zh/antibody/product/Septin-7-Antibody-Polyclonal/PA5-56181), anti-SEPT9 (PA5-100077, https://www.thermofisher.cn/cn/zh/antibody/product/SEPT9-Antibody-Polyclonal/PA5-100077), anti-GSDMD (MA5-44666, https://www.thermofisher.cn/cn/zh/antibody/product/GSDMD-Antibody-clone-PD00-18-Recombinant-Monoclonal/MA5-44666), anti-JNK1/2 (AHO1362, https://www.thermofisher.cn/cn/zh/antibody/product/JNK1-JNK2-Antibody-clone-279Q38-Monoclonal/AHO1362), anti-Phospho-JNK1/2 (700031, https://www.thermofisher.cn/cn/zh/antibody/product/Phospho-JNK1-JNK2-Thr183-Tyr185-Antibody-clone-D12H7L17-Recombinant-Monoclonal/700031), anti-WDTC1 (PA5-113155, https://www.thermofisher.cn/cn/zh/antibody/product/WDTC1-Antibody-Polyclonal/PA5-113155), anti-V5 (AF2894, https://www.beyotime.com/product/AF2894-50%CE%BCl.htm), anti-HA (AF2858, https://www.beyotime.com/product/AF2858-50%CE%BCl.htm), anti-Myc (AF2864, https://www.beyotime.com/product/AF2864-50%CE%BCl.htm), anti-Flag (AF519, https://www.beyotime.com/product/AF519.htm), pan Acetyl-Lysine antibody (AF5632, https://www.beyotime.com/product/AF5632.htm), pan Di-Methyl-Lysine antibody (AF5701, https://www.beyotime.com/product/AF5701.htm), pan Phospho-Serine/Threonine antibody (AF5725, https://www.beyotime.com/product/AF5725.htm)

Eukaryotic cell lines

Policy information about cell lines and Sex and Gender in Research

Cell line source(s)	iBMDMs were kindly provided by Dr. Feng Shao (National Institute of Biological Sciences, Beijing, China). Peritoneal macrophages were isolated from mice. PBMCs were obtained from human blood samples. NIH-3T3 (ATCC, CRL-1658), L929 (ATCC, CCL-1), TC-1 cells (ATCC, CRL-2493) and HEK-293FT cells (Invitrogen, R70007) were purchased from ATCC. Primary cells (peritoneal macrophages) were derived from mice of both sexes (equal distribution). According to the Research-SAGER-guidelines, immortalized cell lines (iBMDMs, iBMDMTet-on SEPT2 shRNA, Atat1-/-, Atat1-/-Scnn1b-/-, Xbp1-/- and HEK-293FT) are not required to state the sex.
Authentication	Cell lines obtained from ATCC have been authenticated using short tandem repeat (STR) analysis.
Mycoplasma contamination	All cells were tested for mycoplasma contamination and the negative cells were used in the study.
Commonly misidentified lines (See ICLAC register)	No commonly misidentified cell lines were used.

Animals and other research organisms

Policy information about studies involving animals; ARRIVE guidelines recommended for reporting animal research, and Sex and Gender in Research

Laboratory animals	WT C57BL/6J mice were purchased from Jackson Laboratory. To generate myeloid-cell-specific SEPT2-deficient (Sept2fl/fl Lyz2-Cre) mice and tamoxifen-inducible SEPT2 conditional knockout (Sept2fl/fl Lyz2-Cre-ERT2) mice, we inter-crossed mice that contained loxP sequence flanking the 4-5 exons of SEPT2 (Sept2fl/fl) with Lyz2-Cre mice or C57BL/6JSmoc-Lyz2em1(2A-CreERT2-WPRE-pA)Smoc mice (Shanghai Model Organisms Center, Inc., Shanghai, China), respectively. HSPA5K327Q mice (B6/JGpt-Hspa5em1(K327Q)/Gpt) were constructed and identified by GemPharmatech Co., Ltd. (Nanjing, China). Referring to previous reports, the K > Q mutation was constructed to mimic acetylation(55, 56). To obtain the p.K327Q point mutation, the nucleic acid change, c.979A>C, was introduced into the endogenous Hspa5 mouse locus using sgRNA (5'-TGTCTTCTCAGCATCAAGCA-3', Chr2: 34774335(+)) and the repair oligonucleotide (5'-AGAAAAGGCTAAGAGAGCCTTGTCTTCTCAGCATCAAGCAAGAATTGAAATTGAGTCCTTCTTCGAAGGAGAAGACTTCTCAGAGACCCTTACTCGGGCCCAATTTGAAGAGCTGAACATGGTATGCTCCTTGACAGTGCTAATGGAATCCGCTTAGACTGTAGAATTTGGGATAACTAAATAAGGTCTGGGTGGTCAGC-3'). The sgRNA, repair oligonucleotide and Cas9 mRNA were pronuclear microinjected into C57BL/6J embryos, which were implanted into pseudo-pregnant females to generate F0 heterozygotes. F0 heterozygotes were inter-crossed with WT mice to generate F1 heterozygotes. Set up the F1 heterozygous×F1 heterozygous cross to obtain F2 homozygous and WT littermates. F2

	homozygous were used for experiments. At the beginning of experiment, indicated numbers of six- to eight-week-old mice were randomly grouped and treated accordingly in each experimental condition.
Wild animals	This study did not involve wild animals.
Reporting on sex	Mice of both sexes (equal distribution) were used for experiments. All mice were age- and sex-matched in each experiment.
Field-collected samples	This study did not involve samples collected from the field.
Ethics oversight	Animal experimental procedures were approved by the Laboratory Animal Welfare and Ethics Committee of Chongqing University.

Note that full information on the approval of the study protocol must also be provided in the manuscript.

Plants

Seed stocks	There are no plants involved in this study
Novel plant genotypes	There are no plants involved in this study
Authentication	There are no plants involved in this study

Flow Cytometry

Plots

Confirm that:

☒ The axis labels state the marker and fluorochrome used (e.g. CD4-FITC).

☒ The axis scales are clearly visible. Include numbers along axes only for bottom left plot of group (a 'group' is an analysis of identical markers).

☒ All plots are contour plots with outliers or pseudocolor plots.

☒ A numerical value for number of cells or percentage (with statistics) is provided.

Methodology

Sample preparation	To analyze the innate immune cell populations in lungs, mice were perfused with sterile PBS and the left lung lobe was digested into single cell suspensions. To analyze the macrophage polarization, PMs obtained from mice were purified using F4/80 antibody. Single cell suspensions and purified PMs were tagged with fluorescently labeled antibodies as the indicated.
Instrument	BD LSR II flow cytometer (BD Biosciences)
Software	FlowJo 10.6.2 and GraphPad Prism 8.3.0
Cell population abundance	PMs obtained from mice were purified using F4/80 antibody.
Gating strategy	The gating strategy of innate immune cell populations in lungs has been provided in the Supplementary Fig. 1h. iNOS, CD80, CD86 and Arg-1, CD163, CD206 were used as M1-like and M2-like macrophage markers, respectively. The gating of iNOS high, CD80 high, CD86 high, Arg-1 high, CD163 high, CD206 high and HSPA5 high populations were determined against those of the uninfected control.

☒ Tick this box to confirm that a figure exemplifying the gating strategy is provided in the Supplementary Information.
